# Dynamical models reveal anatomically reliable attractor landscapes embedded in resting-state brain networks

**DOI:** 10.1162/imag_a_00442

**Published:** 2025-01-24

**Authors:** Ruiqi Chen, Matthew Singh, Todd S. Braver, ShiNung Ching

**Affiliations:** Division of Biology and Biomedical Sciences, Washington University in St. Louis, St. Louis, MO, United States; Department of Electrical and Systems Engineering, Washington University in St. Louis, St. Louis, MO, United States; Department of Psychological and Brain Sciences, Washington University in St. Louis, St. Louis, MO, United States

**Keywords:** resting state fMRI, dynamical systems modeling, individual differences, resting state networks, bifurcations, attractors

## Abstract

Analyses of functional connectivity (FC) in resting-state brain networks (RSNs) have generated many insights into cognition. However, the mechanistic underpinnings of FC and RSNs are still not well-understood. It remains debated whether resting-state activity is best characterized as reflecting noise-driven fluctuations around a single stable state, or instead, as a nonlinear dynamical system with nontrivial attractors embedded in the RSNs. Here, we provide evidence for the latter, by constructing whole-brain dynamical systems models from individual resting-state fMRI (rfMRI) recordings, using the Mesoscale Individualized NeuroDynamic (MINDy) framework. The MINDy models consist of hundreds of neural masses representing brain parcels, connected by fully trainable, individualized weights. We found that our models manifested a diverse taxonomy of nontrivial attractor landscapes including multiple equilibria and limit cycles. However, when projected into anatomical space, these attractors mapped onto a limited set of canonical RSNs, including the default mode network (DMN) and frontoparietal control network (FPN), which were reliable at the individual level. Further, by creating convex combinations of models, bifurcations were induced that recapitulated the full spectrum of dynamics found via fitting. These findings suggest that the resting brain traverses a diverse set of dynamics, which generate several distinct but anatomically overlapping attractor landscapes. Treating rfMRI as a unimodal stationary process (i.e., conventional FC) may miss critical attractor properties and structure within the resting brain. Instead, these may be better captured through neural dynamical modeling and analytic approaches. The results provide new insights into the generative mechanisms and intrinsic spatiotemporal organization of brain networks.

## 1 Introduction

Resting-state fMRI (rfMRI) has become an important tool to probe the link between ongoing brain dynamics and cognition (Lowe, 2012). The most common analytic approach utilized in rfMRI studies is to characterize brain-wide statistical associations (known as functional connectivity or FC) and relate them to cognitive, behavioral, and phenotypic indices (Reinen et al., 2018;Smith et al., 2015). However, the dynamical processes that generate the observed rfMRI fluctuations and statistics (e.g., FC) remain elusive. In particular, it is unknown whether resting-state dynamics can be best described as a unimodal stationary process featuring statistical fluctuations around the mean, or instead, as a nonlinear dynamic system with nontrivial fluctuations associated with stable or metastable patterns deviating from the mean (Laumann et al., 2017;Lurie et al., 2020;Roberts et al., 2019).

The prior FC literature has provided mixed support for both hypotheses. Traditionally, FC is considered to be stationary over the scanning session (Biswal et al., 1995). Correspondingly, the underlying dynamics are found to contain a stable equilibrium (point attractor) at the global mean, and the noisy fluctuations around this stable mean produce the observed FC pattern (Deco, Ponce-Alvarez, et al., 2013). However, recent years have witnessed the rapid development of an analysis technique called time-varying functional connectivity (tvFC), also known as dynamic FC (Lurie et al., 2020). The tvFC method identifies recurring short-time-windowed FC patterns that differ from the mean FC. These transient patterns have been found to be quite reliable within individuals and across populations (Abrol et al., 2017;Choe et al., 2017). More interestingly, transient FC patterns but not the mean FC were found to predict psychopathology (Reinen et al., 2018). These findings seem to suggest the existence of nontrivial, functionally salient fluctuations present in resting state dynamics.

However, the nature and validity of tvFC characterization is itself still under debate. For example, it is known that head motion and physiological noise generate confounds in FC (Power et al., 2012), and even more so for tvFC, which relies on data from shorter duration timeseries (i.e., windowed epochs). More fundamentally, even if tvFC faithfully captures the temporal evolution of neural covariation patterns, it is still unclear whether tvFC states are merely snapshots of the noisy fluctuations around a stable mean, or if they can be associated with nontrivial dynamics. Indeed, tvFC states might be generated from various kinds of nontrivial dynamics (Heitmann & Breakspear, 2018), including multistability (existence of multiple stable states) and metastability (winnerless competition without any stable states) (Roberts et al., 2019). However, an influential paper (Laumann et al., 2017) showed that tvFC clustering can produce very similar outcomes when applied to either real data or stationary noise with matched mean FC and power spectral density. Follow-up studies have tried to resolve this problem using two main approaches: (1) estimating the rate of change around each individual tvFC window in a continuous instead of discrete (as would be in clustering) manner (Battaglia et al., 2020); or (2) defining the states using other statistics, such as phase locking pattern (Vohryzek et al., 2020). While overcoming some drawbacks of tvFC clustering, these methods are still descriptive and thus cannot explain why tvFC arises in the first place. Therefore, to understand the substrate of brain-wide associations, their temporal fluctuations, and ultimately the resting-state dynamics that produce such associations, it is necessary to go beyond descriptive methods and adopt a more mechanistic framework.

Dynamical systems modeling and analysis can provide unique insights into the problem of nontrivial fluctuations in resting state dynamics. Dynamic models of brain activity predict the evolution of activation timeseries given an initial estimate of hidden states. Thus, they provide a generative mechanism for resting-state dynamics and associated statistics, such as tvFC. To date, the evidence for nontrivial fluctuations from dynamic modeling is also mixed (Deco, Ponce-Alvarez, et al., 2013;Nozari et al., 2023;Park et al., 2018;Sip et al., 2023). There are two relevant types of neural models that have been utilized to characterize resting-state brain dynamics as measured by rfMRI: structural-connectome-informed models, and directly-parameterized models. The first type of models usually contain hundreds of sub-components representing brain parcels, connected by weights derived from the brain’s structural connectome, for example, through diffusion tensor imaging. Early models of this type typically included only a few free parameters, such as a global scaling factor for connectivity, that were directly fit to the fMRI data. From these models, it was consistently found that the emergent dynamics involved multiple nontrivial metastable states (Roberts et al., 2019) or attractors (Deco & Jirsa, 2012;Deco, Ponce-Alvarez, et al., 2013), and the distribution of nontrivial attractors has been suggested to reflect the organization of resting-state functional networks (Golos et al., 2015). However, a recent study (Sip et al., 2023) that replaced the neural mass approximation of regional dynamics with a more powerful approximation scheme (i.e., an artificial neural network) reported the opposite, with a single globally stable attractor located at the mean. The second type of models do not assume the structural connectome to be the best surrogate for characterizing functional coupling, but rather directly optimize the effective connectivity between regions by predicting empirical fMRI time series. Most of the existing work adopting this approach has utilized the framework of Dynamical Causal Modeling (DCM;Friston et al., 2003). Although nonlinear DCM has been proposed, it is computationally too expensive for more than 10 nodes (Friston et al., 2019). Therefore, most rfMRI DCMs have used a linear approximation (Razi et al., 2017), which, by definition, cannot express nontrivial fluctuations. It has been argued that such stationary linear models have even lower mean estimation error than common nonlinear models for rfMRI (Nozari et al., 2023). However, a rigorous Bayesian model comparison found that a time-varying (‘dynamic’, short-time-windowed) linear DCM clearly outperformed a stationary linear DCM (Park et al., 2018). In short, previous studies have associated rfMRI with a variety of dynamics ranging from a monostable linear or weakly nonlinear system to a multistable strongly nonlinear system, with diverging evidence for nontrivial fluctuations. What might be the explanation for such inconsistent results?

Here, we suggest that prior approaches have captured some, but not all of the critical aspects of resting-state brain dynamics. We hypothesize that the resting brain is particularly sensitive to modulation, and, as such, can manifest a spectrum of different dynamics that systematically vary across individuals and time. It has been suggested that the resting brain is close to bifurcation, such that a small change in control parameters will alter the stability of the trivial attractor located at the mean (Deco, Ponce-Alvarez, et al., 2013). However, it remains unknown whether both sides of the bifurcation can be observed in the same fMRI dataset, and whether such bifurcations best characterize differences between individuals, or state changes within individuals across different time periods. Previous models have either been too constrained to express diverse sets of dynamics, or lack the specificity to describe individual differences and session-to-session variations. In this project, we overcome these prior limitations by adopting the Mesoscale Individualized NeuroDynamics (MINDy) framework (Singh, Braver, et al., 2020). A key advantage of MINDy models is that they combine the expressiveness of nonlinear neural mass models with the flexibility and individuality of directly parameterized effective connectivity. Our prior work has validated that MINDy models generate individualized, robust, and reliable fits of rfMRI data, with nontrivial dynamics observed (Singh, Braver, et al., 2020). Here, we used the MINDy framework to analyze the taxonomy of resting-state brain dynamics, and to more comprehensively characterize how they change across individuals and time. We fit MINDy models of rfMRI data from over five hundred participants and each of two scanning sessions in the Human Connectome Project (HCP;Smith et al., 2013) to elucidate the dynamic profiles that best explained rfMRI signals. We then analyzed the existence of anatomically reliable attractors and ghost attractors, which are the signatures of a class of bifurcations, showing that the latter frequently occurs. Finally, we show that such attractors were consistent across the population and represent differential activation patterns observed across well-known functional brain networks, such as the default mode network (DMN) and frontoparietal control network (FPN).

## 2 Methods

### 2.1 Data preprocessing

We used the rfMRI data from the HCP Young Adult dataset (Smith et al., 2013). Informed consent was obtained during the original study. Before accessing the HCP data, researchers are required to sign the data use terms (which we did). Data were originally collected on a 3T scanner with a TR of 720 ms and 2 mm isotropic voxels. Participants underwent two scanning sessions on separate days. Each session included two scanning runs of 1200 TRs (around 15 minutes), one using right-to-left phase encoding direction and the other left-to-right. Participants were instructed to stay awake with eyes open and maintain relaxed fixation on a bright cross hair overlaid on a dark background, presented in a darkened room.

We adopted the preprocessing pipeline suggested in (Siegel et al., 2017), which was shown to effectively suppress the influence of head motion in rsFC-behavior associations. Because we were particularly interested in session-to-session variations in dynamics, we used relatively strict inclusion criteria to make sure the data in all runs were sufficiently clean. Specifically, we only included participants with neither missing runs nor runs that had more than 1/3 (400 out of 1200) frames with high head motion (see below), resulting in a total number of 510 participants.

We began with the rfMRI data provided by HCP that had been minimally preprocessed, motion-corrected, and denoised with FIX-ICA (Smith et al., 2013). Following (Siegel et al., 2017), the data were first detrended and then motion scrubbed with framewise displacement (FD) and temporal derivative of variation (DVARS;Afyouni & Nichols, 2018). FD and DVARS were filtered for respiratory artifact with a 40-th order 0.06–0.14 Hz band stop filter. Frames with FD above 0.2 mm or DVARS above 1.05 times of the median were linearly interpolated. We then regressed out from the data the top five principal components of the white matter and the cerebrospinal fluid signals (CompCor), and the mean signal from the gray matter.

After preprocessing, the data were averaged within each parcel according to the 200-parcel atlas from (Schaefer et al., 2018). Data points exceeding 5 standard deviations in each time series were linearly interpolated using MATLAB’s interp1() function to mitigate outlier influence on subsequent preprocessing. To obtain the underlying neural activity, we deconvolved the data with the canonical hemodynamic response function (HRF) from (Friston et al., 1998) using Wiener deconvolution (Wiener, 1949), a deconvolution technique that minimizes the influence of noise. We used a 30-point HRF kernel and a noise-power to signal-power ratio of 0.02. Finally, the data were z-scored within each timeseries.

### 2.2 Model architecture and fitting

We adopted the Mesoscale Individualized NeuroDynamics (MINDy) framework from (Singh, Braver, et al., 2020). A MINDy model contains interconnected neural masses representing brain parcels, with trainable and individualized connection weights. Each neural mass is assumed to follow an S-shape input-output transfer function with a trainable region-specific curvature. Activity decays with a trainable region-specific rate. The ordinary differential equation of the model is thus:

$$\begin{array}{l} \frac{d\text{x}}{dt}(t)=f\left(\text{x}(t)\right)=W\psi_{\alpha }\left(\text{x}(t)\right)-D⊙\text{x}(t)\\ \psi_{\alpha }(x)=\sqrt{\alpha^{2}+{\left(bx+0.5\right)}^{2}}-\sqrt{\alpha^{2}+{\left(bx-0.5\right)}^{2}} \end{array}$$ (1)

Here,$x(t)\in {\mathbb{R}}^{N}$is the neural activity hidden state at timet, whereNis the number of parcels.$W\in {\mathbb{R}}^{N\times N}$is the connectivity matrix. The transfer function$\psi_{\alpha }$is applied element-wise with each region’s respective curvature parameterα.$D\in {\mathbb{R}}^{N}$is the decay, and⊙indicates element-wise multiplication.bis another parameter controlling the shape of the transfer function, currently fixed as20 / 3. To prevent overfitting and improve interpretability, we required the connectivity matrixWto be the sum of a sparse matrix$W_{S}$and a low-rank matrix$W_{L}=W_{1}W_{2}^{T}$, where$W_{1},W_{2}\in {\mathbb{R}}^{N\times k}$andk​<N. Here, we choseN=200(k=72) as it achieves a balance between granularity and computational efficiency. However, we also repeated the analysis withN=100andN=400and obtained very similar attractors.

While the model was specified using continuous-time dynamics, the training data were sampled discretely after every TR (Δt=0.72second). Therefore, it is more convenient to formulate the model as discrete-time dynamics using a first-order approximation:

$$\begin{array}{l} \Delta {\text{x}}_{n}:={\text{x}}_{n+1}-{\text{x}}_{n}\approx f\left({\text{x}}_{n}\right)\Delta t=W_{d}\psi_{\alpha }\left({\text{x}}_{n}\right)-D_{d}⊙{\text{x}}_{n}\\ W_{d}=W\Delta t,D_{d}=D\Delta t \end{array}$$ (2)

Here,${\text{x}}_{n}\;=\text{x}\left(n\Delta t\right)$is then-th sample ofx.$W_{d}$and$D_{d}$are the discrete-time counterparts ofWandDrespectively. With a slight abuse of notation, hereafter we useWandDto refer to$W_{d}$and$D_{d}$(similarly for$W_{1},W_{2},W_{s}$) as the optimization and analysis is performed on the discrete-time model. When comparing the data sampled at different frequencies, the continuous-time dynamics can be recovered from$W_{d}$and$D_{d}$given the TRΔt. It is also worth noting thatΔtis a constant multiplicative factor which will only scale the speed of the dynamics uniformly, without changing the direction of the flow or the location of attractors in state space.

We obtained the hidden statesxfrom the preprocessed rfMRI data for each participant and session, with two runs combined. The ground-truth difference$\Delta {\text{x}}_{n}$was estimated using forward differentiation${\text{x}}_{n+1}-{\text{x}}_{n}$. Since HCP data utilize an exceptionally fast TR, we performed two-point moving average smoothing on the difference to reduce noise, i.e.,$\hat{\Delta }\;{\text{x}}_{n}:=\left({\text{x}}_{n+2}-{\text{x}}_{n}\right)/2$. However, similar results were found even without this smoothing step (see Supplementary Section 8). We optimizedW,αandDto minimize the difference between prediction and ground-truth while enforcing sparsity of the connectivity using L1 regularization (Donoho, 2006). The loss function is defined as:

J=12‖Δ^ xn−[(WS+W1W2T)ψα(xn)−D⊙xn] ‖22  +λ1‖WS‖1+λ2Tr(|WS|)+λ3(‖W1‖1+‖W2‖1) (3)

where$\lambda_{1}=0.075$,$\lambda_{2}=0.2$,$\lambda_{3}=0.05$are the regularization hyperparameters, and$Tr\left(\left|W_{S}\right|\right)$is the absolute sum of the diagonal elements of$W_{S}$. Optimization was performed using gradient descent with Nesterov Accelerated Adaptive Moment Estimation (NADAM;Dozat, 2016). Parameters were updated after each minibatch of 300 random samples. We stopped the training at 5000 minibatches when the test-retest reliability of the parameters started to drop. To prevent the weights from being unnecessarily small due to regularization penalty, we performed an additional global rescaling of the parameters by fitting$\hat{\Delta }\;\text{x}=p_{W}W\psi_{\alpha }(\text{x})-p_{D}D⊙\text{x}$with two scalar parameters$p_{W},p_{D}\in \mathbb{R}$, and factored them intoWandD. It is worth noting that a 200-parcel MINDy model can be fit on a standard laptop within 15 seconds, enabling the analysis of the whole HCP dataset within a reasonable amount of time.

### 2.3 Model simulation and numerical analysis of attractors

For each model, we randomly sampled 1000 frames from the model’s training data as initial conditions for numerical simulation. The dynamics were integrated for 1600 steps with step size equal to one TR, namely${\text{x}}_{n+1}={\text{x}}_{n}+W\psi_{\alpha }\left({\text{x}}_{n}\right)-D⊙{\text{x}}_{n}$. For some models with limit cycles that contained extremely slow ghost attractors, we prolonged the simulation until the state recurred after a full cycle. The stable equilibria were identified as follows: First, we defined a trajectory (simulation) as already converged to a stable equilibrium if$\left|x_{n+1}-x_{n}\right|<10^{-6}$for every parcel and every time pointN−10≤n≤N−1, whereNis the number of simulation time steps. The terminal state${\text{x}}_{N}$from all converged trajectories were then clustered together based on a simple Euclidean distance threshold of0.1. Starting from the first converged trajectory, we computed the distance between the terminal state of each trajectory with already identified equilibria. The state was clustered to a specific identified equilibrium if the distance was smaller than the threshold, otherwise it was selected as a new equilibrium. Similarly, we defined a trajectory as already converged to a stable limit cycle if it approached and then left a small neighborhood of the terminal state$\left\{\text{x}|{\left\|\text{x}-{\text{x}}_{N}\right\|}_{2}<\;\;\;0.5\right\}$at least once. The interval during the last recurrence and the end of the simulation was considered as the period of the limit cycle, and the samples during this period were extracted to represent the limit cycle. We confirmed the validity of the method by visually inspecting the trajectories and identified attractors after dimensionality reduction using Principal Component Analysis (PCA), as depicted inFigures 1and2. After visual inspection, 20 out of 510 participants were excluded from further analysis due to numerical issues in at least one of their models (seeFig. S8for some examples).

**Fig. 1.**
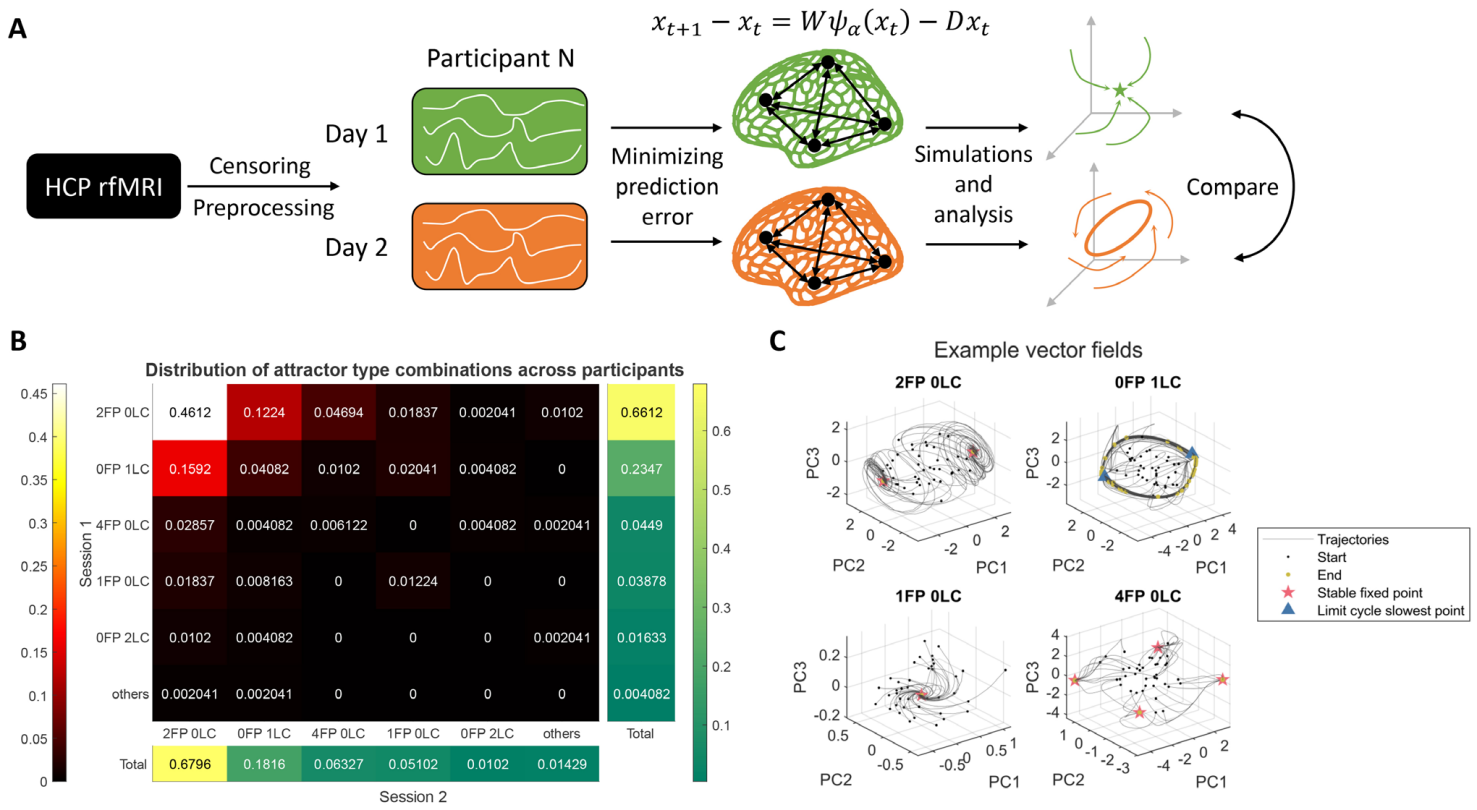
Nonlinear dynamical landscapes underlying individual rfMRI data. (A) Diagram of the analysis pipeline. (B) Distribution of dynamical landscapes across the participants. Numbers indicate the proportion of participants showing certain type of dynamics in session 1 (column index) and session 2 (row index). The types were defined using the number and types of attractors. ‘FP’ indicates stable equilibrium (fixed point), and ‘LC’ indicates stable limit cycle. Types with frequency less than 0.5% were grouped into ‘others’ (seeFig. S11). Marginal distribution for session 1 and session 2 are shown in the panel on the right and at the bottom respectively. (C) Simulated trajectories and obtained attractors from example models, projected onto the first three principal components (PCs) of the trajectories. Black and yellow dots mark the initial and final state of each trajectory. Numerically identified stable equilibria are denoted by red stars. The slowest points on the limit cycles were denoted by blue triangles (seeSection 2). Note that the distribution of final states (yellow dots) on the limit cycle reflects the relative size of the speed. The denser the distribution, the slower the dynamics. We used 1000 simulations per model to identify the attractors but only showed 40 here for visualization.

**Fig. 2.**
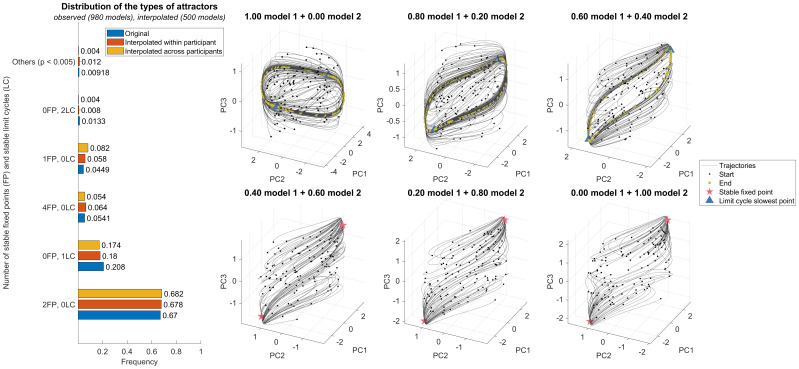
Observed taxonomy of dynamics was consistent with bifurcation-induced continuous spectrum. Left: Distribution of the type of dynamics in linearly interpolated models and empirically fitted models. The interpolated models were obtained by convex combinations of the dynamics from two models from the same participant in different sessions (‘within-participant’) or randomly sampled from all models (‘across-participant’). Right: Bifurcation process between two models of a same HCP participant. Trajectories and attractors were visualized in the same way asFigure 1. Note that the distribution of speed on the limit cycle (indicated by the distribution of final states) became more and more non-uniform as the first model bifurcated towards the second model.

We observed that the distribution of speed on the limit cycles was very non-uniform, sometimes varying by orders of magnitude. Therefore, we selected the slowest point on each limit cycle$\text{arg}\underset{{\text{x}}_{n}}{\;\text{min}}{\left\|{\text{x}}_{n+1}-{\text{x}}_{n}\right\|}_{2}$as the ‘ghost’ attractors. Additionally, for models with one limit cycle, due to the symmetry of the dynamicsf(−x)=−f(x), we added a pair of symmetric slowest points rather than a single one.

### 2.4 Bifurcation analysis

We induced a bifurcation between two models by creating convex combinations of the dynamics. Denoting the two model’s dynamics as$\Delta \text{x}=f_{1}(\text{x})$and$\Delta \text{x}=f_{2}(\text{x})$respectively, we constructed a new model as$\Delta \text{x}=\gamma f_{1}(\text{x})+\left(1-\gamma \right)f_{2}(\text{x})$, where0≤γ≤1is the bifurcation parameter. We considered two ways to combine the observed models. In the within-individual case,$f_{1}$and$f_{2}$were the two models from the two sessions of a same participant. In the across-individuals cases,$f_{1}$and$f_{2}$were sampled from all models. We randomly sampledγfrom the uniform distribution in[0,1]as well as a pair of models for 500 times, and extracted the attractors of these bifurcated models using the numerical method described above. To improve efficiency, here we used 120 random samples from the standard normal distribution as initial conditions for simulation (which was enough to extract all attractors, seeFig. S9). We then characterized the vector field by the number and types (equilibria or limit cycles) of attractors and compared their distribution across the bifurcated models and the fitted models inFigure 2.

### 2.5 Reliability analysis

Because each hidden state dimension in MINDy model represents the activation level of one parcel, each equilibrium state (or ghost attractor) can be understood as a neural activation pattern over the cortex. We quantify the ‘anatomical similarity’ between two such patterns by their Pearson correlation over 200 parcels. As the number of attractors might differ across models, we defined the*dominant attractor similarity (DAS)*between two models as the maximum similarity between their attractors. The distribution of DAS between all pairs of models from different sessions is shown inFigure 3, separated by whether the two models come from a same participant and whether they have same type of dynamics (i.e., same number of stable equilibria and limit cycles).

**Fig. 3.**
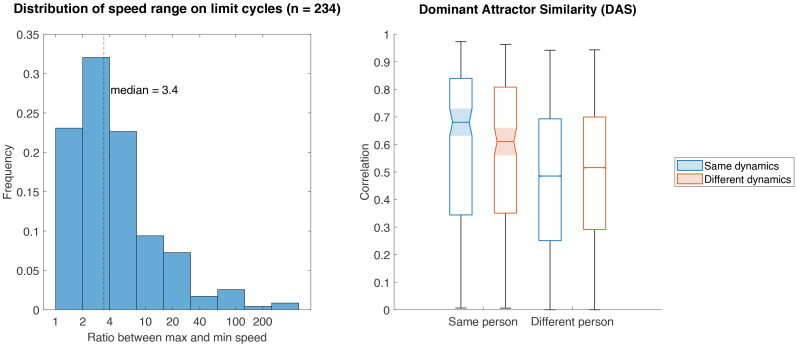
Attractors were more similar within than across individual regardless of changes in dynamical landscapes. Left: Distribution of the speed variability on limit cycles. For each limit cycle in all fitted models, we calculated the ratio between the maximum and minimum speed when the state travels along the limit cycle. Right: Box plot for the distribution of dominant attractor similarity (DAS, seeSection 2.5) between all pairs of models, separated by whether the two models come from a same person and whether they have the same type of dynamics (seeSection 2). Lines on the boxes indicate the maximum, first quartile, median, last quartile, and minimum of each distribution. Tapered, shaded region around the median indicates 95% confidence interval of the median.

### 2.6 Clustering analysis

We usedK-means algorithm to cluster all attractors and ghost attractors across all participants and sessions. We used cosine distanced(x,y)=1−cos∠(x,y)for clustering and scaled each attractor to unit norm for visualization. To assess the robustness of these results, we also tried an alternative clustering method, the robust sparse K-means technique, which deploys Euclidean distance criteria (Brodinová et al., 2019) and conducts variable selection prior to clustering. The number of clustersKis determined by the cluster instability index (Lange et al., 2004) with a candidate list ofKranging from 2 to 10. For eachK, the data were randomly divided into two partitions for 30 iterations. We ran theK-means algorithm on the first partition to obtain the cluster centroids, and used these centroids to classify the samples in the second partition. The classification result was compared to the results of directly runningK-means on the second partition. A misclassification cost was computed after matching the labels using the Hungarian algorithm. This cost is then averaged across the 30 partitions and normalized by the null cost computed in the same way but with random labels, resulting in an instability index for eachK. The local minimum of instability was selected as the number of clusters for the final clustering. We also repeated our analysis using either only the stable equilibria or only the ghost attractors on the limit cycles, and in each case both the clustering instability index (Fig. S13) and cluster centroids (Figs. S15 and S16) were very similar to the results inSection 3.5.

## 3 Results

### 3.1 Model parameters captured reliable individual differences

We obtained 1020 MINDy models, one for each of two rfMRI scanning session associated with 510 HCP participants (Fig. 1A). The models explained about40%of variance in the temporal derivatives of the test set (Fig. S1). To understand the concordance of the models with data, we produced model-generated FC and tvFC by injecting independent white noise to each parcel. In this sense, FC and tvFC arise from the dynamics of the model, rather than its inputs. We found that these model-generated FC and tvFC matched well with those of the data (Fig. S4). The consistency of each parameter set (connectivity, curvature, decay) within individuals and across sessions was around 0.7-0.8. This quantity dropped to around 0.5–0.7 between individuals, indicating that the obtained models were reliable, but also individualized (Fig. S5).

To perform an initial validity check that our models captured meaningful individual differences in the dynamics, we attempted to assess whether our obtained parameters could be connected to individual variation in cognitive measures. We performed Canonical Correlation Analysis (CCA) between the connectivity matrix of the models and the phenotypic measures available in the HCP dataset (Goyal et al., 2020;Smith et al., 2015). Interestingly, we obtained very similar results to (Smith et al., 2015), who used a connectivity matrix obtained via independent components analysis. We identified a single ‘positive-negative mode’ that was significantly correlated between the MINDy connectivity matrix and the behavioral measures, explaining a significant proportion of variance for both. Post-hoc correlation found that this mode was most positively related to fluid intelligence, and most negatively related to substance use (Fig. S6). Therefore, the obtained MINDy models, indeed, capture reliable individual behavioral differences.

### 3.2 rfMRI embeds diverse nonlinear dynamics with nontrivial attractors

We next analyzed the asymptotic behavior of the neural trajectories. For this, we randomly initialized and forward simulated the models to obtain their limit sets and, specifically, reveal any asymptotically stable fixed points and limit cycles. As initially suggested in (Singh, Braver, et al., 2020), the dynamics captured in our obtained MINDy models were highly nonlinear, with nontrivial attractors and limit cycles found in the large majority of cases (Fig. 1C). Only around 5% of models exhibited a single stable equilibrium at the origin (Fig. 1B). It is worth noting that the observed nontrivial dynamics cannot be simply attributed to any bias in our method, because MINDy models will actually recover trivial dynamics when fit on the noisy simulations of a stable linear system (Fig. S2). Most importantly, MINDy also correctly produced a globally attractive origin, rather than nontrivial attractors, when it was fit on noise data with covariance and mean spectral power density that matched real data (Fig. S2). On the contrary, it is known that standard tvFC methods cannot disambiguate such noise and actual data (Laumann et al., 2017). Our findings, thus, support an interpretation of time-varying rfMRI activity being most appropriately described as emanating from a nonlinear dynamical system.

### 3.3 Induced bifurcations explained the heterogeneity of observed dynamics

Interestingly, although the obtained MINDy parameters were very reliable within each individual across sessions (Fig. S5), the number or type (fixed point or limit cycle) of attractors were still different in about half of all participants (Fig. 1B). Such finding is nontrivial because the attractor landscape topology is a very high-level feature of the models, and thus usually more reliable than the underlying parameters. For example, the toy model shown inFigure S12always has two pairs of equilibria for any parameter0<μ<1, and always has one stable limit cycle for anyμ>1. It is only when the dynamics are close to a so-called bifurcation point (μ=1) that a small change in the model parameters can result in a topological discontinuity of the dynamical landscape. Such bifurcating dynamics were, indeed, implied to be present in the brain (Cocchi et al., 2017;Deco & Jirsa, 2012). Therefore, we hypothesized that the resting-state dynamics might be close to bifurcation and thus better described by a spectrum of possible dynamics that are sampled at each session, rather than a single monolithic brain state. To test the hypothesis, we constructed a parameterized ‘interpolated’ model as a convex combination of obtained models (i.e.,$(\gamma ){\text{ Model}}_{1}$+$(1-\gamma ){\text{ Model}}_{2}$), either within each participant (between the two models) or across all participants. If this hypothesis is correct, by varyingγ, it should be possible to induce bifurcations that result in similar taxonomy of dynamics. We analyzed the dynamics of the interpolated models and compared the distribution of the number and types of attractors with those of the original fitted models (Fig. 2). The taxonomy of dynamics was very similar for fitted models and interpolated models, consistent with the idea that the dynamics we obtained are a reflection of whole-brain dynamics undergoing bifurcations between topologically distinct vector fields. We also performed another similar analysis by directly mixing the weights of different models instead of interpolating the dynamics, and obtained qualitatively similar results. The spectrum of dynamics remained statistically indistinguishable from the observed spectrum when mixing the weights of the two models from the same participant, and only changed quantitatively but not qualitatively when mixing weights across the population. On the contrary, shuffling the weights across entries destroyed the structure of the weights and reduced all models to monostable trivial dynamics (Supplementary Fig. S3).

### 3.4 Individualized attractors were reliable despite changes in dynamics

Having established that the observed dynamics can be understood as bifurcating across a continuous spectrum, it is natural to ask how the brain can maintain stable or consistent function if the underlying dynamics are constantly changing. We, thus, hypothesized that there must be some anatomical commonality between the different obtained dynamics, leading to consistency that would be observable in terms of whole-brain activation patterns.

To probe this question, we looked for aspects of the dynamics that were relatively invariant to the bifurcations we observed. Most notably, we found that when the dynamics differed across sessions for a same person, they most commonly switched between having two equilibria or having one limit cycle (Fig. 1B). This is consistent with the well-known infinite-period bifurcation (Keener, 1981).Figure S12presents an analytic toy model for infinite-period bifurcation with one parameterμ. If the mean value ofμis far from the bifurcation pointμ=1, the attractor landscape topology is relatively insensitive toμ(e.g.,μ=5results in a same topology withμ=3). It is only whenμis close to the bifurcation point that a small perturbation ofμ(e.g., due to noise in estimation) can lead to both limit cycle and point attractor dynamics observed. An infinite-period bifurcation begins with a limit cycle that contains a ghost attractor. As the bifurcation parameter changes, the ghost attractor becomes ‘infinitely slow’ (i.e., neural activity lingers near it for long periods of time) until eventually the bifurcation occurs and a stable node (along with an saddle node) gets created out of it. In our data, when limit cycles were observed, the distribution of speed along them was highly non-uniform, sometimes varying by orders of magnitude (Fig. 3, left). In our interpolated models, this form of bifurcation, indeed, occurs (Fig. 2, right panel). Therefore, we hypothesize that the ghost attractors and point attractors provide a set of stable ‘operating points’ for the changing dynamics during rest.

If the session-to-session variability in fitted dynamics can be explained by such a bifurcation, we would expect that ghost attractors should be close to the stable equilibria observed in the other session. We defined ghost attractors as the slowest point on each limit cycle, and calculated the anatomical similarity (i.e., Pearson correlation across 200 dimensions/parcels) between all (true and ghost) attractors. Because the number of attractors might differ across sessions, we defined the*dominant attractor similarity (DAS)*to be the maximum correlation over all pairs of attractors from the two models under consideration (seeSection 2). The DAS was higher within subject versus across subjects (Fig. 3, right), even when restricting the former to models showing different types of dynamics (e.g., two equilibria or one limit cycle) and the latter to models showing same type of dynamics (the second and the third boxes in the middle inFig. 3, right). Therefore, our results support the hypothesis that the resting brain is bifurcating between different dynamics, while maintaining a set of reliable attractors as stable operating points.

### 3.5 Attractors aligned with canonical resting state networks across population

Next, we examined whether these attractors could be interpreted from a functional standpoint. Interestingly, the DAS was high (around 0.5) even between different participants (Fig. 3), indicating the existence of consistent patterns across the whole population. Therefore, we clustered the attractors across all participants and sessions, where the number of clustersKwere selected according to the cluster instability index (Lange et al., 2004, seeSection 2,Fig. S13). We obtained near perfect cluster stability only forK=4, apart from the less interesting solutionK=2. Note that the attractors always exist in pairs (seeSection 2), soK=2represents only one pattern and its opposite (Fig. S14).

**Fig. 4.**
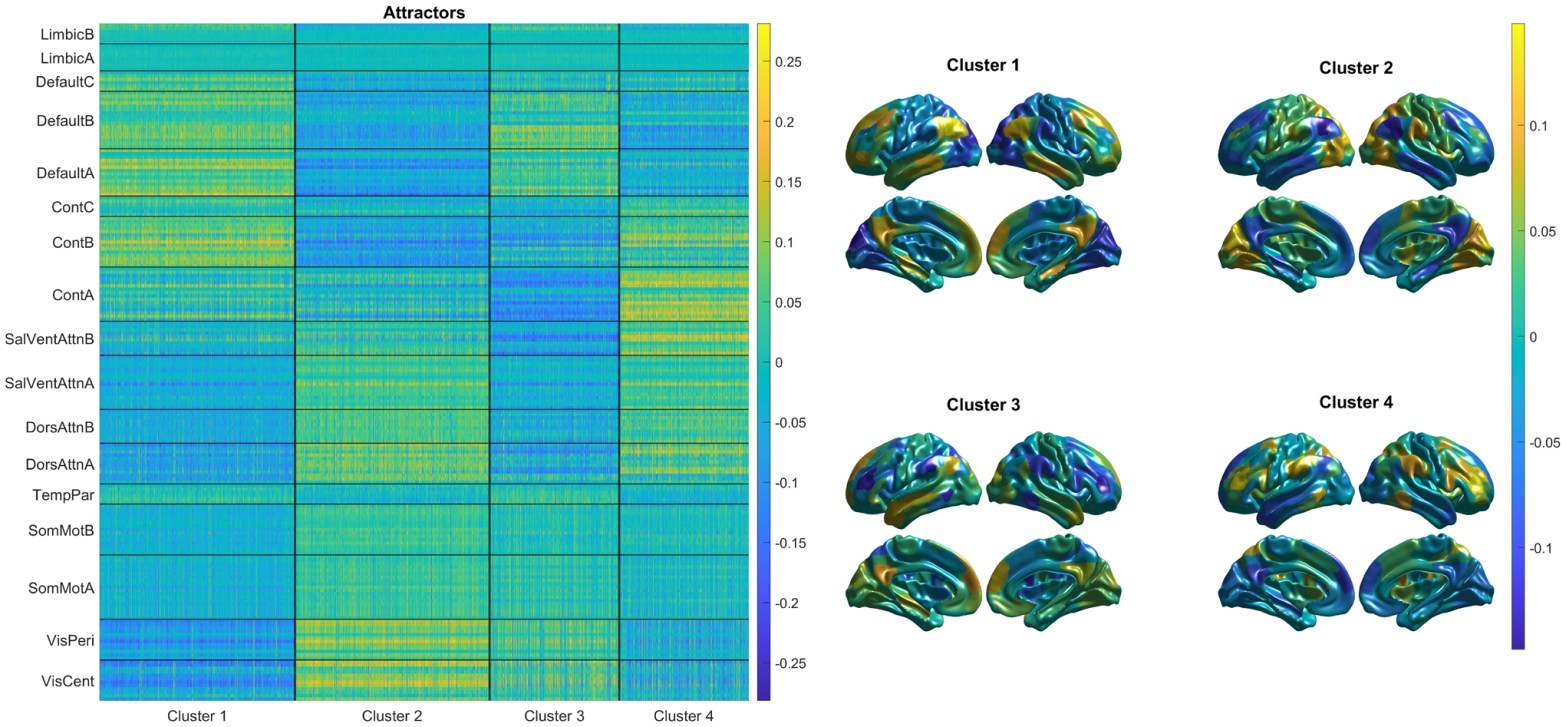
Attractors aligned with canonical resting-state networks across the population. Left: All stable equilibria and ghost attractors. Each row corresponds to one parcel and each column corresponds to one attractor. Rows are sorted according to the (Schaefer et al., 2018) atlas and columns are sorted according to cluster assignments. Thick horizontal and vertical lines separate the functional networks and clusters, respectively. Attractors are scaled to unit norm for visualization purpose. Right: cluster centroids visualized as the activation patterns over the cortex.

The individual attractors within each cluster aligned very well with the cluster centers (Fig. 4). Even more interestingly, the activation patterns were highly modular, respecting the functional network organization defined in the (Schaefer et al., 2018) atlas, even though the model fitting process was completely agnostic to parcel labeling. In particular, one large cluster (Cluster 1) was dominated by the activation of DMN and FPN. Another cluster was dominated by the FPN and dorsal/ventral attention networks (Cluster 4). The other two clusters showed the opposite activation profiles (i.e., Cluster 2 opposite to Cluster 1, Cluster 3 opposite to Cluster 4). Note that these clustering results were robust to the use of an alternative clustering method which employs variable selection prior to clustering (seeSection 2andFig. S17).

The clusters and network organization in combination accounted for more than 40% of the total variation across all participants, sessions, and parcels (Fig. S18). The activation pattern was better explained by functional network organization rather than the spatial proximity of parcels on the cortical surface (Fig. S19). Furthermore, the attractor clusters emerged regardless of whether we only included the equilibria, the limit cycle ghost attractors, or both (Figs. S15 and S16), supporting the hypothesis that they represent the reliable activation dynamics present in resting-state networks.

## 4 Discussion

In this study, we adopted a nonlinear dynamical systems modeling framework to analyze how resting state brain dynamics varied across individuals and time. We found that resting-state brain activity embedded diverse nonlinear dynamics that included nontrivial attractors rather than a single globally stable equilibrium at the mean. Interestingly, the dynamics reliably varied between individuals. Moreover, instead of being stationary, the dynamics underwent bifurcations across different scanning sessions even within the same individual. Furthermore, the observed spectrum of dynamics could be fully recovered through induced bifurcations between fitted models. Consistent with such bifurcations, the attractors and ghost attractors were anatomically reliable within and between individuals. These attractors were organized into distinct clusters that reflected the activation of different functional brain networks, particularly the DMN, the FPN, and the dorsal/ventral attention networks. Using the formal language of dynamical systems and bifurcation theory, our results shed light on the connection between nontrivial fluctuations in resting state activity, the organization of functional brain networks, and stable individual differences. We provide a modeling and analysis framework that describes the individualized nontrivial dynamics in rfMRI, while maintaining interpretability and tractability. A variety of dynamical features can be derived from the models for future brain-phenotype association studies. Our results also enable model-based interventions into brain dynamics (e.g., via noninvasive neurostimulation), which might hold great potential for individualized treatments of brain disorders and even cognitive enhancement (Singh et al., 2022).

### 4.1 On the role of directly-parameterized dynamical models in rfMRI analysis

Many different methods have been developed to probe the existence of non-trivial patterns in rfMRI, ranging from clustering-based methods (Reinen et al., 2018;Vohryzek et al., 2020), graph-theory-based methods (Diez & Sepulcre, 2018), to dynamical-model-based methods (Deco & Jirsa, 2012;Razi et al., 2017). One important distinction between such methods is whether they are*descriptive*or*generative*(Friston, 2011). Descriptive methods identify meaningful features (e.g., attractor-like patterns) directly from data. Generative methods (e.g., MINDy), on the contrary, rely on a model that predicts (generates) new samples of the data itself, and then extracts meaningful features from that model. While relying on more assumptions, generative methods also possess unique advantages. The use of a model allows the features to be extracted in a more rigorous and reliable way. In this paper, we revealed the presence of attractors according to their strict mathematical definition, by using a simple and replicable numerical procedure, without relying on surrogate measures such as cluster centroids of tvFC. More importantly, unlike descriptive methods, generative methods illuminate not only*what*these features look like, but also*how*they emerge. In this paper, we revealed the modular (i.e., network-based) organization and structure of cortical activation patterns that have been discovered using descriptive methods (Yeo et al., 2011). However, unlike previous studies, we showed for the first time that such organization patterns emerge from nontrivial attractor landscapes generated by nonlinear interactions occurring between parcels. Beyond analysis of*existing*data, generative models also enable analysis of unforeseen dynamics through perturbations of parameters. For example, we revealed the bifurcating nature of the dynamics by interpolating across models identified for each person and session. A more extensive exploration of the dynamical repertoire could lead to new mechanistic insights about different functional or pathological brain states (McIntosh & Jirsa, 2019). Last but not least, generative models can also produce ground-truth data without confounds (e.g., head motion) to help develop statistical testing framework for descriptive methods, which is crucial for establishing the validity of such methods (Lurie et al., 2020).

Among generative dynamical models, another important distinction lies between structural-connectivity-informed models (Breakspear, 2017) and directly-parameterized models (Razi et al., 2017) such as MINDy. The connection weights in the former type of models reflect the structural connectivity (SC) between nodes (i.e., cortical parcels), usually approximated as being proportional to the number of streamlines that can be reconstructed between them from diffusion tensor imaging (DTI) data. On the contrary, the connection weights of the latter type of models are the effective connectivity (EC) strengths, which reflect the functional influence of one node on another (Friston, 2011), and which are optimized to fit the fMRI data. It is clear that SC and EC (as well as FC) are all closely related, yet distinct (Friston, 2011;Suárez et al., 2020). This is because it is not a requirement that EC reflects the monosynaptic anatomic connections that are modeled by SC. More generally, the relationship between EC and SC will be mediated by the type of synaptic connections (O’Rourke et al., 2012) and the ratio of axons targeting excitatory (v.s. inhibitory) neurons (Somogyi et al., 1998). A useful way to describe the distinction between SC-based and EC-based models might be in terms of the balance between biological realism and functional phenomenology (Bassett et al., 2018). With SC-based models, the goal is often to explore the functionality that emerges from realistic, anatomical constraints, while in EC-based models it is to reveal how certain observed functions may depend on specific organizational structures. Because we aimed to analyze the dynamics underlying experimentally recorded resting-state brain activity, we adopted the EC-based framework but retained a certain level of biological realism by defining the physical brain regions as network nodes. We chose to utilize this approach as it is currently quite challenging to obtain reliable estimates of individual SC parameters (Maier-Hein et al., 2017), and a key aspect of our research endeavor is to build individualized models. Conversely, using population-level SC parameters to build individualized models would significantly constrain the repertoire of dynamics that can possibly emerge (Deco, Ponce-Alvarez, et al., 2013). Therefore, the EC-based framework is more appropriate for the questions of interest in this paper.

### 4.2 Nontrivial attractors and resting state networks

Resting-state brain activity is traditionally treated as a unimodal stationary process fluctuating around a stable mean. However, recent studies have begun to explore nontrivial recurring patterns in resting-state dynamics (Abreu et al., 2020;Liu & Duyn, 2013;Roberts et al., 2019;Vohryzek et al., 2020). Most notably, time-varying functional connectivity (tvFC, also called ‘dynamic’ FC) studies have suggested that the resting brain traverses multiple states associated with distinct brain-wide association patterns (Lurie et al., 2020). However, it is debated whether these recurring patterns, indeed, represent nontrivial fluctuations, or merely reflect snapshots of the trivial fluctuations around the mean (Laumann et al., 2017). Due to these challenges, it has been questioned whether the new insights we gain from these techniques are relatively marginal compared to the increased difficulties of analysis and interpretation (Nozari et al., 2023).

We argue that controversies regarding the presence of non-trivial fluctuations reflect the lack of mechanistic interpretations that can be derived from short-time-windowed methods typically utilized to estimate tvFC. Dynamic-systems-based modeling provides a more generative explanation for the observed dynamics and statistics. Phase portraits of the fitted models reveal insights about the resting brain dynamics without sacrificing the ease and interpretability of analysis. Here, by using a nonlinear, individualized, and fully trainable dynamical systems modeling framework, we provide evidence for the presence of nontrivial fluctuations in rfMRI dynamics. Most importantly, when fitted on stationary noise with FC and a mean power spectrum that match real data, our model correctly recreated a monostable dynamic system, while tvFC has been demonstrated to produce spurious nontrivial states in this situation (Laumann et al., 2017). Therefore, the nontrivial attractors consistently observed in our fitted models are less likely to be explained by a bias inherent in the method. Compared to other modeling studies, our model relies on fewer assumptions about the nature of connectivity patterns (Deco, Ponce-Alvarez, et al., 2013;Sip et al., 2023). Specifically, instead of assuming that streamline density (i.e., an estimate of SC) is a good surrogate of functional coupling at the mesoscale level, which has been challenged by some studies (Barttfeld et al., 2015), we posit a ‘sparse plus low rank’ structure with all the weights directly optimized toward empirical fMRI timeseries. Very interestingly, even without prior constraints on the functional network structure, the attractors that emerged from the models still exhibited highly modular activation patterns that respect the organization of functional networks. Such convergence of evidence is further supported by the fact that, within MINDy, there is a high degree of similarity between the structure of the weight matrix between localized parcels (i.e., estimated EC), and that which has been discovered for ICA-defined distributed brain network (i.e., estimated FC), despite using very different node types and metrics of connectivity. This is evidenced by the highly similar information found in MINDy regarding cognitive individual differences as revealed by CCA, that replicates prior FC-based findings (Fig. S6). Furthermore, unlike most previous work that has relied upon population-level models, we were able to show that the identified nontrivial attractors are not only consistent across the population, but also test-retest reliable within each individual. Therefore, our results suggest that resting brain dynamics might be supported by the interaction between noise and nontrivial attractor landscapes. Conversely, it is interesting that we did not observe metastable dynamics in our models, which are dynamics that do not contain any attractors, but instead reflect a set of nontrivial competing patterns. Metastable dynamics can generate time-varying activation patterns even in the absence of noise and have been found in macroscopic brain models (Roberts et al., 2019). This distinction might arise from the differences between the SC-based models utilized in prior work (Roberts et al., 2019) and the EC-based models adopted here.

It has long been hypothesized that nontrivial attractors represent ‘functional’ states that can be spontaneously traversed during rest, as if exploring the repertoire of operating points (Deco, Jirsa, & McIntosh, 2013). Although there has been some theoretical work focused on the functional relevance of resting state attractors (Kurikawa & Kaneko, 2013), empirical evidence has been scarce. Our work supports such theoretical accounts, by showing that nontrivial resting state attractors reflect selective activation of functional brain networks, and contain reliable individual differences. It will, thus, be very interesting to extend the MINDy framework to examine task as well as resting states, in order to analyze how such nontrivial attractors might contribute to cognitive computations. Similar to DCM methods (Friston et al., 2019), to extend the MINDy framework to task states, we can couple the recurrent dynamics with an input term representing task control signals. Control-theoretic based analyses can provide a useful tool to characterize the interaction between task demands and the inherent dynamics associated with attractor landscapes. Such analyses can potentially shed light on how the resting brain ‘prepares’ stable motifs for cognitive computation, advancing of our understanding of the mechanistic link between the resting state and cognition.

### 4.3 Bifurcations and the critical brain

We presented three lines of evidence that resting-state brain dynamics are not only nontrivial, but also bifurcating. First, the topology of the dynamic landscapes changed across sessions even when the controlling parameters remained highly reliable, consistent with the definition of bifurcations. Second, we induced bifurcations between fitted models and recovered the full spectrum of the dynamics observed, confirming that the fitted models can be understood as samples from such a continuous spectrum encompassing several bifurcations. Third, we identified anatomically reliable attractors or ghost attractors on a cycle, consistent with the prediction of an infinite-period bifurcation. Our results, thus, provide a richer description of both the invariants and changes in resting-state dynamics, showing that even though the statistical outputs (FC) of two datasets might seem similar, they could be supported by distinct but still intimately related dynamics.

The most interesting conclusion from our analysis is that the resting-state brain is highly sensitive to modulation, in which a small perturbation can bifurcate it toward various different dynamics. Such a scenario is statistically rare because the dynamics (more specifically, the attractor landscape topology) are very high-level features of the model, and thus usually more reliable than the underlying parameters (e.g.,Fig. S12). To illustrate this point, we first showed that even though the correlation between the parameters of any two models was high (around 0.6), we still observed at least nine different kinds of dynamics, in the sense of different numbers or types of attractors. Moreover, we found that although the parameter correlation within each participant was even higher (around 0.75), the dynamics were still different between sessions for almost half of all participants.

We suggest that the sensitivity of resting brain dynamics could provide a potential mechanism for cognitive flexibility, as the brain can easily bifurcate from the resting state toward various different dynamics that might be advantageous for different cognitive computations. If that were the case, the task state brain dynamics should be more rigid than resting state, and should vary across tasks according to the computation required. In line with this idea, it was found that task-driven input reduced the variability of whole-brain dynamics compared to rest (Ito et al., 2020;Ponce-Alvarez et al., 2015). Further, the global connectivity of the frontoparietal network was found to systematically vary across 64 tasks, with more similar connectivity for tasks that require more similar computation (Cole et al., 2013). Most interestingly, it has been found that the similarity between rsFC and task-specific FC positively correlated with task performance across multiple tasks, and the similarity between rsFC and task-general FC can be related to fluid intelligence (Schultz & Cole, 2016), indicating that cognitive ability is related to how efficiently resting-state dynamics can transform into a variety of different task dynamics. Our study provides a more mechanistic framework that can capture the changes in the spectrum of generative dynamics, not just the statistics of such dynamics (i.e., FC). Extending the MINDy framework to task contexts will, thus, provide a strong test for such hypothesis.

Our results also relate to the idea of criticality in the brain. Note that the word ‘criticality’ has been used in at least two different ways in neuroscience. In the broad sense, criticality refers to the emergence of slow, large-amplitude (scale-free) fluctuations in a dynamical system that is close to losing its stability, without specifying the kind of instability to which it is transformed (Cocchi et al., 2017). In a narrow sense, such transformations are restricted to those occurring between an ordered state (stability) and an unordered state (chaos;O’Byrne & Jerbi, 2022). Our results support criticality of rfMRI dynamics in the broad sense rather than the narrower sense. We found that our models show either a stable low-activity state with no nontrivial attractors, or an unstable low-activity state with nontrivial attractors, suggesting the existence of supercritical bifurcations where a high-activity attractor emerged ‘above’ the low-activity attractor as the latter loses its stability. Such bifurcations have been shown to give rise to slow, scale-free fluctuations (Cocchi et al., 2017). In previous models of whole-brain dynamics, it has been found that the criticality associated with such bifurcations improved the response sensitivity to external stimuli (Deco, Jirsa, & McIntosh, 2013). However, critical dynamics are not always optimal for all tasks. For example, the sensitivity to inputs also reduces the reliability of the response (Fagerholm et al., 2015). Therefore, it is proposed that instead of staying critical, brain dynamics should reverberate between multiple regimes near criticality (Wilting et al., 2018). Our results support this hypothesis with novel evidence that the brain traverses near criticality, potentially balancing the computational advantages of each regime across different computations.

If the resting brain resides in this critical regime that can be easily modulated into different dynamics, one interesting question is how the brain might implement such modulation to utilize different dynamics. On a longer timescale, such as the dynamics we obtained here across a 30-minute resting-state scan, neuromodulator systems might be the best candidate. It is well established that neuromodulators can affect brain connectivity and whole-brain dynamics (Shafiei et al., 2019;Shine et al., 2018). The arousal system might play a particularly important role in the fluctuations of resting-state dynamics (Laumann et al., 2017). On a shorter timescale, such as during the execution of cognitive tasks, goal-directed top-down modulation might also bifurcate the dynamics. Theoretically, such bifurcations might relate to the proactive control mode in the dual mechanism framework for cognitive control (Braver, 2012). In contrast to reactive control, proactive control refers to the active maintenance of goal-related information and the biasing of cognitive computations even before cognitively demanding events occur. In parallel, goal-directed bifurcation might transform the dynamical landscape to bias the neural processing even before receiving cognitive inputs. Therefore, combining MINDy modeling with cognitive tasks as well as neuromodulatory manipulations holds great potential to further our understanding about the dynamical flexibility of the brain.

### 4.4 Limitations and future directions

In this study, we characterized the variable nature of resting-state dynamics by comparing across participants and scanning sessions. Such variation inevitably interweaves with measurement and modeling error, as the uncertainty in parameters can lead to different types of attractor landscapes, particularly if they are close to bifurcation. However, importantly, the main finding in this paper is not in defining a specific number of equilibria or limit cycles that are postulated to exist in the resting brain, but rather the opposite, that is, a demonstration that resting brain dynamics are close to bifurcation, such that the number of equilibria and limit cycles might change from time to time. Nevertheless, the problem remains as to whether the observed bifurcations are induced by ‘true’ physiological changes or noise. This question can be addressed via two future directions of research. First, the temporal non-stationarity of brain activity dynamics can be explored using data with higher temporal resolution (e.g., M/EEG) and longer duration, so as to reduce the influence of test-retest errors. Second, the functional relevance of different attractor landscapes under various task (vs. rest) settings can be directly examined. A stronger test for the non-stationarity of resting-state dynamics would require comparing the dynamics across different long temporal epochs within a single scanning run. Such analysis is difficult to apply within datasets such as the HCP, which only has 15-minute long scans, roughly as much as the data needed to obtain a reliable MINDy model (Singh, Braver, et al., 2020). However, such analyses would be possible with datasets having at least 30 contiguous minutes of rfMRI data, such as the Midnight Scanning Club (Gordon et al., 2017). Another possibility is to apply the MINDy framework to EEG or MEG datasets (Singh et al., 2023), as these have much lower dimensionality and much higher sampling rate. It might be even possible to update the model parameters in real time for EEG/MEG data (Singh et al., 2023).

There are also limitations associated with the MINDy model used in the analysis. The current MINDy models do not include an intercept/bias term, and the dynamics are anti-symmetric with respect to the neural hidden states (seeSection 2). Such assumptions were made according to the excitation-inhibition-balance principle (van Vreeswijk & Sompolinsky, 1996) and have been adopted in resting-state DCM studies too (Razi et al., 2017). In such models, the zero vector (corresponding to the mean of the data) is always an equilibrium (though not necessarily stable) and nontrivial equilibria (if any) must exist in pairs. Extending MINDy to task contexts by introducing a task-related input can break such symmetry. Another limitation of the current method is the ability to account for the variations in hemodynamics. Here, we estimated the hidden neural states by a noise-aware deconvolution of BOLD signal with the canonical haemodynamic response function (HRF;Friston et al., 1998). However, it has been suggested that HRF varies significantly across brain regions and individuals (Aguirre et al., 1998). Although MINDy parameters have been demonstrated to be robust against HRF variations (Singh, Braver, et al., 2020), it is unclear how much the emergent dynamics will be influenced, given the observation that the dynamics were sensitive to parameters. Extending our analysis with region-specific HRF (Singh, Wang, et al., 2020) is a natural step to follow. Another direction is to use a biologically more detailed regional dynamics model (Friston et al., 2019). Currently, the recurrent dynamics within each brain region is modeled as a simple self-excitation (or inhibition) with an exponential decay. Despite being computationally more tractable, such a model might not capture the full dynamics within a region, especially the interactions between local sub-populations (Singh et al., 2023). Multi-scale modeling and EEG-fMRI data fusion might be a possible way to improve biological specificity while maintaining computational efficiency. Lastly, while the MINDy framework is specified as a continuous-time dynamical system, the parameter optimization relies on time discretization. In particular, the derivative estimates might be less accurate for data sampled at longer TRs. In the current study, we obtained similar results with or without two-point derivative smoothing (which effectively doubled the TR to 1.44 seconds), indicating that our method should be suitable for most fMRI datasets with fast-to-moderate TR. For datasets with even longer TR, it might be helpful to adopt higher-order ODE solvers instead of Euler discretization, despite sacrificing computational efficiency.

From a methodological perspective, it will be interesting to conduct a more systematic comparison between the dynamics identified through MINDy and other modeling methods. For example, a question of interest is whether it is possible to reveal nontrivial attractor landscapes using other types of nonlinear effective-connectivity-based models (e.g.,Friston et al., 2019). Also relevant is to identify the potential conditions under which a structural-connectome-based model will manifest nontrivial dynamics (Deco, Ponce-Alvarez, et al., 2013) versus not manifesting them (Sip et al., 2023). From a cognitive neuroscience perspective, the current study demonstrates how the novel lens of dynamical systems and attractor landscapes can be utilized for theory and analysis regarding the relationship between intrinsic dynamics (i.e., present during resting states) and the characteristics of task-related cognitive computations. An important direction for future studies is to test for associations between individualized resting-state dynamical motifs and cognitive traits and task performance. This could be accomplished using traditional correlational analysis, or perhaps more interestingly, by adapting the MINDy framework to analyze how dynamics and attractors change between resting and task states. As a mechanistic alternative to FC and tvFC analysis, our framework can also generate new insights into the dynamical changes associated with different dimensions of cognitive variation, including states of consciousness (e.g., sleep, meditation, psychedelics), developmental stages, or dysfunction associated with psychiatric and neurological disorders.

## Supplementary Material

Supplementary Material

PCSurf_dW_00_0FP_1LC

PCSurf_dW_00_2FP_0LC

PCSurf_dW_10_2FP_1LC

PCSurf_dW_20_4FP_0LC

## Data Availability

The rfMRI data are available athttps://humanconnectome.org/study/hcp-young-adult/overview. The preprocessing scripts are available athttps://github.com/rq-Chen/Singh2020PreProc. The analysis scripts are available athttps://github.com/rq-Chen/MINDy_rfMRI_stable. The standalone MINDy modeling toolbox is available athttps://github.com/singhmf/MINDy/tree/master/MINDy_Base_v1.0.

## References

[b1] Abreu , R. , Jorge , J. , Leal , A. , Koenig , T. , & Figueiredo , P. ( 2020 ). EEG microstates predict concurrent fMRI dynamic functional connectivity states . Brain Topography , 34 ( 1 ), 41 – 55 . 10.1007/S10548-020-00805-1 33161518

[b2] Abrol , A. , Damaraju , E. , Miller , R. L. , Stephen , J. M. , Claus , E. D. , Mayer , A. R. , & Calhoun , V. D. ( 2017 ). Replicability of time-varying connectivity patterns in large resting state fMRI samples . NeuroImage , 163 , 160 – 176 . 10.1016/j.neuroimage.2017.09.020 28916181 PMC5775892

[b3] Afyouni , S. , & Nichols , T. E. ( 2018 ). Insight and inference for DVARS . NeuroImage , 172 , 291 – 312 . 10.1016/j.neuroimage.2017.12.098 29307608 PMC5915574

[b4] Aguirre , G. K. , Zarahn , E. , & D’Esposito , M. ( 1998 ). The variability of human, BOLD hemodynamic responses . NeuroImage , 8 ( 4 ), 360 – 369 . 10.1006/NIMG.1998.0369 9811554

[b5] Barttfeld , P. , Uhrig , L. , Sitt , J. D. , Sigman , M. , Jarraya , B. , & Dehaene , S. ( 2015 ). Signature of consciousness in the dynamics of resting-state brain activity . Proceedings of the National Academy of Sciences , 112 ( 3 ), 887 – 892 . 10.1073/pnas.1418031112 PMC4311826 25561541

[b6] Bassett , D. S. , Zurn , P. , & Gold , J. I. ( 2018 ). On the nature and use of models in network neuroscience . Nature Reviews Neuroscience , 19 ( 9 ), 566 – 578 . 10.1038/s41583-018-0038-8 30002509 PMC6466618

[b7] Battaglia , D. , Boudou , T. , Hansen , E. C. A. , Lombardo , D. , Chettouf , S. , Daffertshofer , A. , McIntosh , A. R. , Zimmermann , J. , Ritter , P. , & Jirsa , V. ( 2020 ). Dynamic functional connectivity between order and randomness and its evolution across the human adult lifespan . NeuroImage , 222 , 117156 . 10.1016/j.neuroimage.2020.117156 32698027

[b8] Biswal , B. , Zerrin Yetkin , F. , Haughton , V. M. , & Hyde , J. S. ( 1995 ). Functional connectivity in the motor cortex of resting human brain using echo-planar MRI . Magnetic Resonance in Medicine , 34 ( 4 ), 537 – 541 . 10.1002/MRM.1910340409 8524021

[b9] Braver , T. S. ( 2012 ). The variable nature of cognitive control: A dual mechanisms framework . Trends in Cognitive Sciences , 16 ( 2 ), 106 – 113 . 10.1016/j.tics.2011.12.010 22245618 PMC3289517

[b10] Breakspear , M. ( 2017 ). Dynamic models of large-scale brain activity . Nature Neuroscience , 20 ( 3 ), 340 – 352 . 10.1038/nn.4497 28230845

[b11] Brodinová , Š. , Filzmoser , P. , Ortner , T. , Breiteneder , C. , & Rohm , M. ( 2019 ). Robust and sparse k-means clustering for high-dimensional data . Advances in Data Analysis and Classification , 13 ( 4 ), 905 – 932 . 10.1007/s11634-019-00356-9

[b12] Choe , A. S. , Nebel , M. B. , Barber , A. D. , Cohen , J. R. , Xu , Y. , Pekar , J. J. , Caffo , B. , & Lindquist , M. A. ( 2017 ). Comparing test-retest reliability of dynamic functional connectivity methods . NeuroImage , 158 , 155 – 175 . 10.1016/j.neuroimage.2017.07.005 28687517 PMC5614828

[b13] Cocchi , L. , Gollo , L. L. , Zalesky , A. , & Breakspear , M. ( 2017 ). Criticality in the brain: A synthesis of neurobiology, models and cognition . Progress in Neurobiology , 158 , 132 – 152 . 10.1016/j.pneurobio.2017.07.002 28734836

[b14] Cole , M. W. , Reynolds , J. R. , Power , J. D. , Repovs , G. , Anticevic , A. , & Braver , T. S. ( 2013 ). Multi-task connectivity reveals flexible hubs for adaptive task control . Nature Neuroscience , 16 ( 9 ), 1348 – 1355 . 10.1038/nn.3470 23892552 PMC3758404

[b15] Deco , G. , & Jirsa , V. K. ( 2012 ). Ongoing cortical activity at rest: Criticality, multistability, and ghost attractors . Journal of Neuroscience , 32 ( 10 ), 3366 – 3375 . 10.1523/JNEUROSCI.2523-11.2012 22399758 PMC6621046

[b16] Deco , G. , Jirsa , V. K. , & McIntosh , A. R. ( 2013 ). Resting brains never rest: Computational insights into potential cognitive architectures . Trends in Neurosciences , 36 ( 5 ), 268 – 274 . 10.1016/j.tins.2013.03.001 23561718

[b17] Deco , G. , Ponce-Alvarez , A. , Mantini , D. , Romani , G. L. , Hagmann , P. , & Corbetta , M. ( 2013 ). Resting-state functional connectivity emerges from structurally and dynamically shaped slow linear fluctuations . Journal of Neuroscience , 33 ( 27 ), 11239 – 11252 . 10.1523/JNEUROSCI.1091-13.2013 23825427 PMC3718368

[b18] Diez , I. , & Sepulcre , J. ( 2018 ). Neurogenetic profiles delineate large-scale connectivity dynamics of the human brain . Nature Communications , 9 ( 1 ), 1 – 10 . 10.1038/s41467-018-06346-3 PMC6155203 30250030

[b19] Donoho , D. L. ( 2006 ). For most large underdetermined systems of linear equations the minimal l1-norm solution is also the sparsest solution . Communications on Pure and Applied Mathematics , 59 ( 6 ), 797 – 829 . 10.1002/CPA.20132

[b20] Dozat , T. ( 2016 ). Incorporating Nesterov momentum into Adam . Proceedings of the 4th International Conference on Learning Representations , pp. 1 – 4 . 10.1109/icaect57570.2023.10117792

[b21] Fagerholm , E. D. , Lorenz , R. , Scott , G. , Dinov , M. , Hellyer , P. J. , Mirzaei , N. , Leeson , C. , Carmichael , D. W. , Sharp , D. J. , Shew , W. L. , & Leech , R. ( 2015 ). Cascades and cognitive state: Focused attention incurs subcritical dynamics . Journal of Neuroscience , 35 ( 11 ), 4626 – 4634 . 10.1523/JNEUROSCI.3694-14.2015 25788679 PMC4363389

[b22] Friston , K. J. , Fletcher , P. , Josephs , O. , Holmes , A. , Rugg , M. D. , & Turner , R. ( 1998 ). Event-related fMRI: Characterizing differential responses . NeuroImage , 7 ( 1 ), 30 – 40 . 10.1006/NIMG.1997.0306 9500830

[b23] Friston , K. J. , Harrison , L. , & Penny , W. ( 2003 ). Dynamic causal modelling . NeuroImage , 19 ( 4 ), 1273 – 1302 . 10.1016/S1053-8119(03)00202-7 12948688

[b24] Friston , K. J. , Preller , K. H. , Mathys , C. , Cagnan , H. , Heinzle , J. , Razi , A. , & Zeidman , P. ( 2019 ). Dynamic causal modelling revisited . NeuroImage , 199 , 730 – 744 . 10.1016/j.neuroimage.2017.02.045 28219774 PMC6693530

[b25] Friston , K. J. ( 2011 ). Functional and effective connectivity: A review . Brain Connectivity , 1 ( 1 ), 13 – 36 . 10.1089/brain.2011.0008 22432952

[b26] Golos , M. , Jirsa , V. , & Daucé , E. ( 2015 ). Multistability in large scale models of brain activity . PLoS Computational Biology , 11 ( 12 ), e1004644 . 10.1371/JOURNAL.PCBI.1004644 26709852 PMC4692486

[b27] Gordon , E. M. , Laumann , T. O. , Gilmore , A. W. , Newbold , D. J. , Greene , D. J. , Berg , J. J. , Ortega , M. , Hoyt-Drazen , C. , Gratton , C. , Sun , H. , Hampton , J. M. , Coalson , R. S. , Nguyen , A. L. , McDermott , K. B. , Shimony , J. S. , Snyder , A. Z. , Schlaggar , B. L. , Petersen , S. E. , Nelson , S. M. , & Dosenbach , N. U. ( 2017 ). Precision functional mapping of individual human brains . Neuron , 95 ( 4 ), 791 – 807.e7 . 10.1016/J.NEURON.2017.07.011 28757305 PMC5576360

[b28] Goyal , N. , Moraczewski , D. , Bandettini , P. , Finn , E. S. , & Thomas , A. ( 2020 ). Computationally replicating the Smith et al. (2015) positive-negative mode linking functional connectivity and subject measures . bioRxiv . 10.1101/2020.04.23.058313

[b29] Heitmann , S. , & Breakspear , M. ( 2018 ). Putting the “dynamic” back into dynamic functional connectivity . Network Neuroscience , 2 ( 2 ), 150 – 174 . 10.1162/netn_a_00041 30215031 PMC6130444

[b30] Ito , T. , Brincat , S. L. , Siegel , M. , Mill , R. D. , He , B. J. , Miller , E. K. , Rotstein , H. G. , & Cole , M. W. ( 2020 ). Task-evoked activity quenches neural correlations and variability across cortical areas . PLoS Computational Biology , 16 ( 8 ), e1007983 . 10.1371/journal.pcbi.1007983 32745096 PMC7425988

[b31] Keener , J. P. ( 1981 ). Infinite period bifurcation and global bifurcation branches . SIAM Journal on Applied Mathematics , 41 ( 1 ), 127 – 144 . 10.1137/0141010

[b32] Kurikawa , T. , & Kaneko , K. ( 2013 ). Embedding responses in spontaneous neural activity shaped through sequential learning . PLoS Computational Biology , 9 ( 3 ), e1002943 . 10.1371/JOURNAL.PCBI.1002943 23505355 PMC3591288

[b33] Lange , T. , Roth , V. , Braun , M. L. , & Buhmann , J. M. ( 2004 ). Stability-based validation of clustering solutions . Neural Computation , 16 ( 6 ), 1299 – 1323 . 10.1162/089976604773717621 15130251

[b34] Laumann , T. O. , Snyder , A. Z. , Mitra , A. , Gordon , E. M. , Gratton , C. , Adeyemo , B. , Gilmore , A. W. , Nelson , S. M. , Berg , J. J. , Greene , D. J. , McCarthy , J. E. , Tagliazucchi , E. , Laufs , H. , Schlaggar , B. L. , Dosenbach , N. U. F. , & Petersen , S. E. ( 2017 ). On the stability of BOLD fMRI correlations . Cerebral Cortex , 27 ( 10 ), 4719 – 4732 . 10.1093/cercor/bhw265 27591147 PMC6248456

[b35] Liu , X. , & Duyn , J. H. ( 2013 ). Time-varying functional network information extracted from brief instances of spontaneous brain activity . Proceedings of the National Academy of Sciences , 110 ( 11 ), 4392 – 4397 . 10.1073/pnas.1216856110 PMC3600481 23440216

[b36] Lowe , M. J. ( 2012 ). The emergence of doing “nothing” as a viable paradigm design . NeuroImage , 62 ( 2 ), 1146 – 1151 . 10.1016/j.neuroimage.2012.01.014 22245648

[b37] Lurie , D. J. , Kessler , D. , Bassett , D. S. , Betzel , R. F. , Breakspear , M. , Kheilholz , S. , Kucyi , A. , Liégeois , R. , Lindquist , M. A. , McIntosh , A. R. , Poldrack , R. A. , Shine , J. M. , Thompson , W. H. , Bielczyk , N. Z. , Douw , L. , Kraft , D. , Miller , R. L. , Muthuraman , M. , Pasquini , L. , … Calhoun , V. D. ( 2020 ). Questions and controversies in the study of time-varying functional connectivity in resting fMRI . Network Neuroscience , 4 ( 1 ), 30 – 69 . 10.1162/netn_a_00116 32043043 PMC7006871

[b38] Maier-Hein , K. H. , Neher , P. F. , Houde , J.-C. , Côté , M.-A. , Garyfallidis , E. , Zhong , J. , Chamberland , M. , Yeh , F.-C. , Lin , Y.-C. , Ji , Q. , Reddick , W. E. , Glass , J. O. , Chen , D. Q. , Feng , Y. , Gao , C. , Wu , Y. , Ma , J. , He , R. , Li , Q. , … Descoteaux , M. ( 2017 ). The challenge of mapping the human connectome based on diffusion tractography . Nature Communications , 8 ( 1 ), 1349 . 10.1038/s41467-017-01285-x PMC5677006 29116093

[b39] McIntosh , A. R. , & Jirsa , V. K. ( 2019 ). The hidden repertoire of brain dynamics and dysfunction . Network Neuroscience , 3 ( 4 ), 994 – 1008 . 10.1162/NETN_A_00107 31637335 PMC6777946

[b40] Nozari , E. , Bertolero , M. A. , Stiso , J. , Caciagli , L. , Cornblath , E. J. , He , X. , Mahadevan , A. S. , Pappas , G. J. , & Bassett , D. S. ( 2023 ). Macroscopic resting-state brain dynamics are best described by linear models . Nature Biomedical Engineering 8 ( 1 ), 68 – 84 . 10.1038/s41551-023-01117-y PMC11357987 38082179

[b41] O’Byrne , J. , & Jerbi , K. ( 2022 ). How critical is brain criticality? Trends in Neurosciences , 45 ( 11 ), 820 – 837 . 10.1016/j.tins.2022.08.007 36096888

[b42] O’Rourke , N. A. , Weiler , N. C. , Micheva , K. D. , & Smith , S. J. ( 2012 ). Deep molecular diversity of mammalian synapses: Why it matters and how to measure it . Nature Reviews Neuroscience , 13 ( 6 ), 365 – 379 . 10.1038/nrn3170 22573027 PMC3670986

[b43] Park , H.-J. , Friston , K. J. , Pae , C. , Park , B. , & Razi , A. ( 2018 ). Dynamic effective connectivity in resting state fMRI . NeuroImage , 180 , 594 – 608 . 10.1016/j.neuroimage.2017.11.033 29158202 PMC6138953

[b44] Ponce-Alvarez , A. , He , B. J. , Hagmann , P. , & Deco , G. ( 2015 ). Task-driven activity reduces the cortical activity space of the brain: Experiment and whole-brain modeling . PLoS Computational Biology , 11 ( 8 ), e1004445 . 10.1371/journal.pcbi.1004445 26317432 PMC4552873

[b45] Power , J. D. , Barnes , K. A. , Snyder , A. Z. , Schlaggar , B. L. , & Petersen , S. E. ( 2012 ). Spurious but systematic correlations in functional connectivity MRI networks arise from subject motion . NeuroImage , 59 ( 3 ), 2142 – 2154 . 10.1016/j.neuroimage.2011.10.018 22019881 PMC3254728

[b46] Razi , A. , Seghier , M. L. , Zhou , Y. , McColgan , P. , Zeidman , P. , Park , H.-J. , Sporns , O. , Rees , G. , & Friston , K. J. ( 2017 ). Large-scale DCMs for resting-state fMRI . Network Neuroscience , 1 ( 3 ), 222 – 241 . 10.1162/NETN_a_00015 29400357 PMC5796644

[b47] Reinen , J. M. , Chén , O. Y. , Hutchison , R. M. , Yeo , B. T. T. , Anderson , K. M. , Sabuncu , M. R. , Öngür , D. , Roffman , J. L. , Smoller , J. W. , Baker , J. T. , & Holmes , A. J. ( 2018 ). The human cortex possesses a reconfigurable dynamic network architecture that is disrupted in psychosis . Nature Communications , 9 ( 1 ), 1157 . 10.1038/s41467-018-03462-y PMC5861099 29559638

[b48] Roberts , J. A. , Gollo , L. L. , Abeysuriya , R. G. , Roberts , G. , Mitchell , P. B. , Woolrich , M. W. , & Breakspear , M. ( 2019 ). Metastable brain waves . Nature Communications , 10 ( 1 ), 1 – 17 . 10.1038/s41467-019-08999-0 PMC6401142 30837462

[b49] Schaefer , A. , Kong , R. , Gordon , E. M. , Laumann , T. O. , Zuo , X.-N. , Holmes , A. J. , Eickhoff , S. B. , & Yeo , B. T. T. ( 2018 ). Local-global parcellation of the human cerebral cortex from intrinsic functional connectivity MRI . Cerebral Cortex , 28 ( 9 ), 3095 – 3114 . 10.1093/CERCOR/BHX179 28981612 PMC6095216

[b50] Schultz , D. H. , & Cole , M. W. ( 2016 ). Higher intelligence is associated with less task-related brain network reconfiguration . Journal of Neuroscience , 36 ( 33 ), 8551 – 8561 . 10.1523/JNEUROSCI.0358-16.2016 27535904 PMC4987432

[b51] Shafiei , G. , Zeighami , Y. , Clark , C. A. , Coull , J. T. , Nagano-Saito , A. , Leyton , M. , Dagher , A. , & Mišić , B. ( 2019 ). Dopamine signaling modulates the stability and integration of intrinsic brain networks . Cerebral Cortex , 29 ( 1 ), 397 – 409 . 10.1093/cercor/bhy264 30357316 PMC6294404

[b52] Shine , J. M. , van den Brink , R. L. , Hernaus , D. , Nieuwenhuis , S. , & Poldrack , R. A. ( 2018 ). Catecholaminergic manipulation alters dynamic network topology across cognitive states . Network Neuroscience , 2 ( 3 ), 381 – 396 . 10.1162/netn_a_00042 30294705 PMC6145851

[b53] Siegel , J. S. , Mitra , A. , Laumann , T. O. , Seitzman , B. A. , Raichle , M. , Corbetta , M. , & Snyder , A. Z. ( 2017 ). Data quality influences observed links between functional connectivity and behavior . Cerebral Cortex , 27 ( 9 ), 4492 – 4502 . 10.1093/cercor/bhw253 27550863 PMC6410500

[b54] Singh , M. F. , Braver , T. S. , Cole , M. , & Ching , S. ( 2023 ). Precision data-driven modeling of cortical dynamics reveals idiosyncratic mechanisms of canonical oscillations . bioRxiv. 10.1101/2023.11.14.567088 PMC11761305 39823302

[b55] Singh , M. F. , Braver , T. S. , Cole , M. W. , & Ching , S. ( 2020 ). Estimation and validation of individualized dynamic brain models with resting state fMRI . NeuroImage , 221 , 117046 . 10.1016/j.neuroimage.2020.117046 32603858 PMC7875185

[b56] Singh , M. F. , Cole , M. W. , Braver , T. S. , & Ching , S. ( 2022 ). Developing control-theoretic objectives for large-scale brain dynamics and cognitive enhancement . Annual Reviews in Control , 54 , 363 – 376 . 10.1016/j.arcontrol.2022.05.001 PMC10798814 38250171

[b57] Singh , M. F. , Wang , A. , Braver , T. S. , & Ching , S. ( 2020 ). Scalable surrogate deconvolution for identification of partially-observable systems and brain modeling . Journal of Neural Engineering , 17 ( 4 ), 046025 . 10.1088/1741-2552/aba07d 32590377 PMC7946334

[b58] Sip , V. , Hashemi , M. , Dickscheid , T. , Amunts , K. , Petkoski , S. , & Jirsa , V. ( 2023 ). Characterization of regional differences in resting-state fMRI with a data-driven network model of brain dynamics . Science Advances , 9 ( 11 ), eabq7547 . 10.1126/sciadv.abq7547 36930710 PMC10022900

[b59] Smith , S. M. , Beckmann , C. F. , Andersson , J. , Auerbach , E. J. , Bijsterbosch , J. , Douaud , G. , Duff , E. , Feinberg , D. A. , Griffanti , L. , Harms , M. P. , Kelly , M. , Laumann , T. , Miller , K. L. , Moeller , S. , Petersen , S. , Power , J. , Salimi-Khorshidi , G. , Snyder , A. Z. , Vu , A. T. , … Glasser , M. F. ( 2013 ). Resting-state fMRI in the Human Connectome Project . NeuroImage , 80 , 144 – 168 . 10.1016/j.neuroimage.2013.05.039 23702415 PMC3720828

[b60] Smith , S. M. , Nichols , T. E. , Vidaurre , D. , Winkler , A. M. , Behrens , T. E. J. , Glasser , M. F. , Ugurbil , K. , Barch , D. M. , Van Essen , D. C. , & Miller , K. L. ( 2015 ). A positive-negative mode of population covariation links brain connectivity, demographics and behavior . Nature Neuroscience , 18 ( 11 ), 1565 – 1567 . 10.1038/nn.4125 26414616 PMC4625579

[b61] Somogyi , P. , Tamás , G. , Lujan , R. , & Buhl , E. H. ( 1998 ). Salient features of synaptic organisation in the cerebral cortex . Brain Research Reviews , 26 ( 2 ), 113 – 135 . 10.1016/S0165-0173(97)00061-1 9651498

[b62] Suárez , L. E. , Markello , R. D. , Betzel , R. F. , & Misic , B. ( 2020 ). Linking structure and function in macroscale brain networks . Trends in Cognitive Sciences , 24 ( 4 ), 302 – 315 . 10.1016/j.tics.2020.01.008 32160567

[b63] van Vreeswijk , C. , & Sompolinsky , H. ( 1996 ). Chaos in neuronal networks with balanced excitatory and inhibitory activity . Science , 274 ( 5293 ), 1724 – 1726 . 10.1126/science.274.5293.1724 8939866

[b64] Vohryzek , J. , Deco , G. , Cessac , B. , Kringelbach , M. L. , & Cabral , J. ( 2020 ). Ghost attractors in spontaneous brain activity: Recurrent excursions into functionally-relevant BOLD phase-locking states . Frontiers in Systems Neuroscience , 14 , 20 . 10.3389/fnsys.2020.00020 32362815 PMC7182014

[b65] Wiener , N. ( 1949 ). Extrapolation, interpolation, and smoothing of stationary time series: With engineering applications . The MIT Press . 10.7551/mitpress/2946.001.0001

[b66] Wilting , J. , Dehning , J. , Pinheiro Neto , J. , Rudelt , L. , Wibral , M. , Zierenberg , J. , & Priesemann , V. ( 2018 ). Operating in a reverberating regime enables rapid tuning of network states to task requirements . Frontiers in Systems Neuroscience , 12 , 55 . 10.3389/fnsys.2018.00055 30459567 PMC6232511

[b67] Yeo , B. T. T. , Krienen , F. M. , Sepulcre , J. , Sabuncu , M. R. , Lashkari , D. , Hollinshead , M. , Roffman , J. L. , Smoller , J. W. , Zöllei , L. , Polimeni , J. R. , Fischl , B. , Liu , H. , & Buckner , R. L. ( 2011 ). The organization of the human cerebral cortex estimated by intrinsic functional connectivity . Journal of Neurophysiology , 106 ( 3 ), 1125 – 1165 . 10.1152/jn.00338.2011 21653723 PMC3174820

