# Supplementary material for: Dynamical models reveal anatomically reliable attractor landscapes embedded in resting-state brain networks
Source: Imaging Neurosci (Camb). 2025 Jan 24;3:imag_a_00442. doi: 10.1162/imag_a_00442 (PMC12140615; doi:10.1162/imag_a_00442)
Supplement: Supplementary Material [file imag_a_00442-supp.pdf]

1 Validation of MINDy modeling

1.1 Goodness of fit

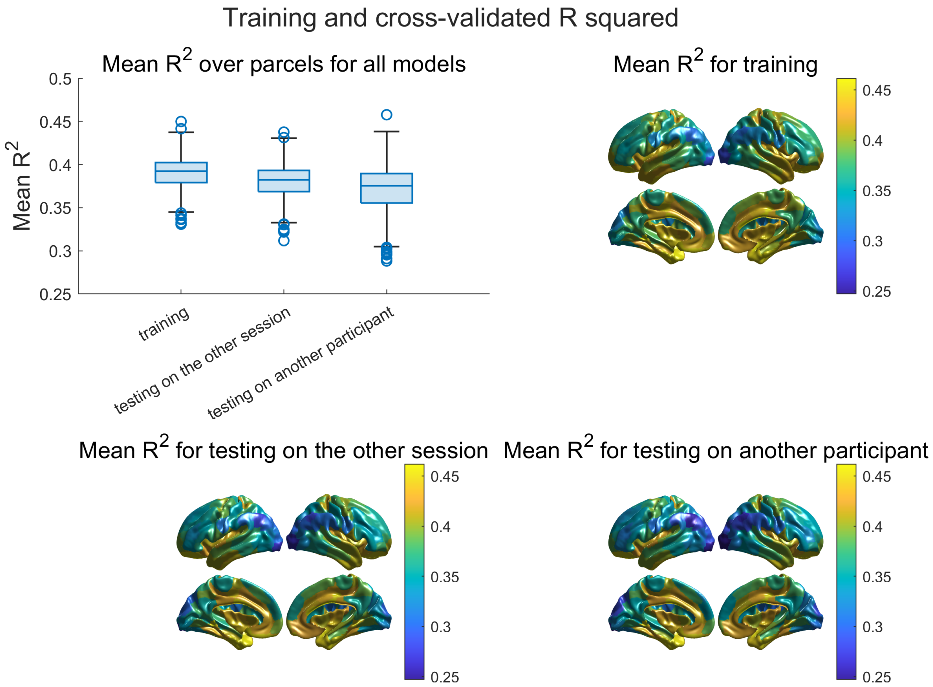

Figure S1: **Goodness-of-fit and cross validation accuracy.** Top-left: distribution of the mean R squared for all models when predicting the training data, the data from the same participant in the other session, or the data from another randomly selected participant. Top-right: Distribution of mean R squared over all models when testing on training data. Bottom-left and bottom-right: similar plots for testing on the other session within participant, or testing on another participant's data.

We tested the prediction accuracy of the fitted models in three cases: the training data, the data from the same participant but in the other session, and the data from a random participant in the same session. The R squared was around 0.4 for all three cases, with the training accuracy being the highest, the within-person transfer being the second and across-person transfer being the third (Figure S1). Therefore, our models indeed captured the individuality of the dynamics.

1.2 Surrogate data simulations

To make sure that the nontrivial dynamics observed in fitted models were not simply due to methodological bias, we investigated whether MINDy can correctly capture the trivial dynamics in closely matched

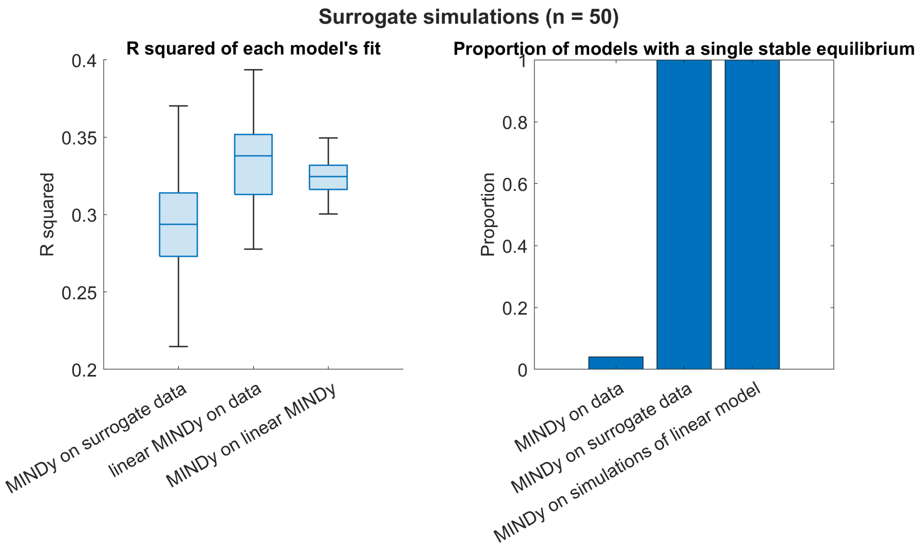

Figure S2: **Fitting MINDy on surrogate data revealed trivial dynamics.** Left: R squared for fitting MINDy on surrogate data; fitting 'linear MINDy' on rfMRI data; and fitting MINDy on the noisy simulations of 'linear MINDy' models. Right: the proportion of models showing a single stable equilibrium (at the origin), for MINDy models fitted on rfMRI data, on surrogate data, and on the noisy simulations of 'linear MINDy' models.

surrogate datasets. We tried two different surrogate simulation schemes and fit MINDy on these simulations using the same hyperparameters as in the main text. The first method follows (Laumann et al., 2017), generating a dataset from noise (without any dynamics) but preserves the covariance (thus FC) and mean power spectrum of the rfMRI data. In (Laumann et al., 2017), it was found that the tvFC method generated indistinguishable results for real data and such surrogate data. However, our model correctly produced a monostable dynamic system without nontrivial fluctuations (Figure S2). The second method fit MINDy on the noisy simulations of a closely-matched linear system. This linear system, referred to as 'linear MINDy', replaces the nonlinear activation function of MINDy with its best linear approximation. The 'linear MINDy' model was fit to rfMRI data using the same hyperparameters and loss function as actual MINDy, thus capturing the statistics of the data and also maintaining the 'sparse plus low-rank' connectivity structure. In fact, we found that the connectivity and decay parameters of 'linear MINDy' is highly correlated with the actual MINDy model fitted on the same data. After fitting the linear model, we simulated the model with additive noise. The magnitude of the noise was set to the root of mean squared error during the fitting of the linear model. The noisy simulations thus represent a close match of the true dataset but generated from inherently linear (and monostable) dynamics. We then fit (nonlinear) MINDy models on these simulations, and the models correctly reproduced a monostable dynamic system with no nontrivial attractors (Figure S2).

1.3 Comparison with weight-shuffled models

To demonstrate that the observed spectrum of dynamics reflects nontrivial structure of the data and does not emerge purely due to estimation error, we compared the spectrum of dynamics of fitted models against that of weight-shuffled models. We selected the fitted models for the first 50 participants and both sessions (100 models in total), then permuted the weights of the models in three different ways: (1) "across cells within model", where we shuffled the entries of each set of the weights (i.e., connectivity matrix, decay, and curvature) in each model. Such permutation destroyed the structure of the weights and was expected to lead to unrealistic dynamics; (2) "within cell across sessions", where we shuffled the weights entry by entry across the two models of the same participant. Namely, shuffled entries of the new weight matrices were selected randomly from the corresponding entries of the two models, thus preserving the structure of the weights while mimicking the effect of estimation error across sessions; (3) "Within cell across population", which is similar to (2) but now selecting weights from all 100 models instead of from only the models from the same participant. We varied the proportion of shuffled weights from 0% (fitted models) to 100%.

Results were shown in Figure S3. For the "across cells within model" condition, the proportion of weights shuffled significantly interacted with the distribution of dynamics ($\chi^2(25) = 453.049$, $p < 0.001$), with a moderate effect size (Cramer's $V = 0.389$). Note that shuffling 20% of weights already eliminated two commonly observed type of dynamics (four fixed points and two limit cycles), and shuffling more than 40% of weights reduced all the models to the monostable trivial dynamics. Such result showed that the observed spectrum of nontrivial dynamics indeed required certain weight structure, which was destroyed after permutation. For the "within cell across sessions" condition, the interaction between proportion of weights shuffled and distribution of dynamics was not significant ($\chi^2(25) = 18.011$, $p = 0.842$, Cramer's $V = 0.077$). Even when we select each entry of the weight matrices randomly from both models of the same participant (i.e., at the level of 100%), we still observed very similar distribution of dynamics, indicating that the weight structure was well preserved across different models of the same participant. Similarly, for the "within cell across population" condition, although the interaction between proportion of weights shuffled and distribution of dynamics was significant ($\chi^2(25) = 53.854$, $p < 0.001$), the effect size (Cramer's $V = 0.134$) was much smaller than in the "across cells within model" condition. In particular, even at the level of 100% shuffling, where each model was created by randomly mixing the corresponding weights of all models, we still observed qualitatively similar spectrum of dynamics. Together with the experiments in main Figure 2, these results suggest that the resting brain indeed manifests several distinct types of nontrivial dynamics that were consistent across the population and more so within person, and MINDy was able to recapitulate such diversity in resting brain dynamics.

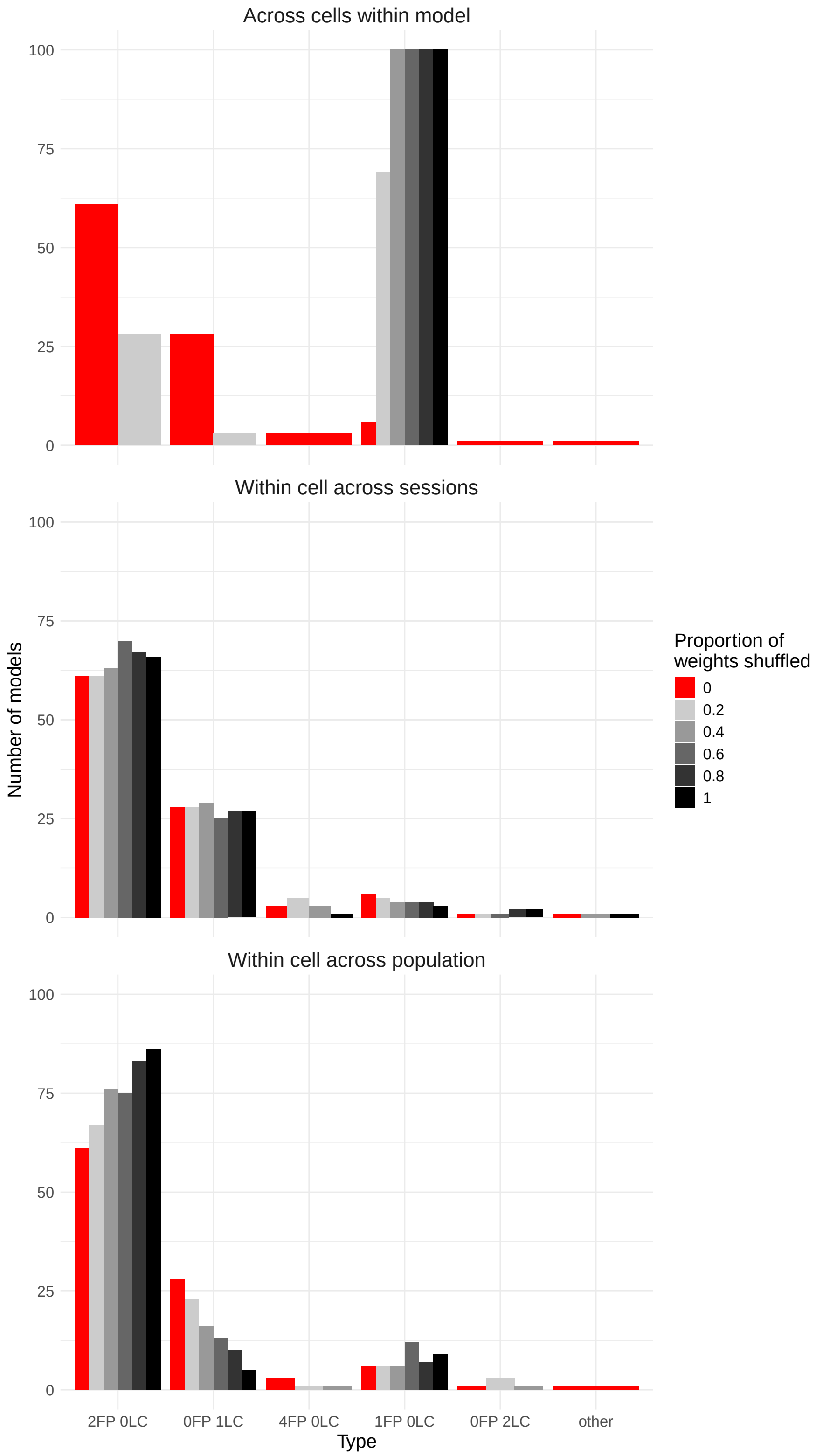

Figure S3: Distribution of the type of dynamics across fitted and shuffled models.

1.4 Dynamical features captured by MINDy simulation

(Singh et al., 2020) found that noisy simulation of MINDy models captured the individual differences in the variance and excursion of tvFC of rfMRI data. Here we demonstrate that MINDy noisy simulation also captures the 'tvFC states'. We simulated the models from the first 30 participants with additive Gaussian white noise. The magnitude of the noise is the root mean squared error during MINDy fitting multiplied by a scaling factor that is constant across all participants. We optimized this factor to minimize the discrepancy between mean FC of the simulation and the data. We searched for the optimal value between 0.1 and 1 using MATLAB's `fminbnd()` function. The optimal scaling turned out to be around 0.45. Because we only fit one more parameter that is the same across all models, this should not lead to significant overfitting. Importantly, noise was added independently for each parcel, thus the correlation structure of the simulated data must emerge from the dynamics instead of the additive noise. We calculated the windowed FC matrices on both the simulations and the data that the models were trained on, with a window size of 53 seconds (following Battaglia et al., 2020) and no overlapping between windows. We then clustered all windowed FC matrices across all models. The cluster centroids were shown in Figure S4. The results were very similar from both real data (lower triangle of each panel) and MINDy simulation (upper triangle), indicating that MINDy can recover the key statistical features of the training data.

2 MINDy parameter reliability and association with behavioural & biophysical measures

2.1 MINDy parameters were individualized and consistent across the population

We characterized the consistency of model parameters across all participants, as well as within each participant. We computed the correlation of each set of parameters (connectivity, curvature or decay) between each pair of models. For each participant, we quantified the similarity between their two models as well as the mean similarity between their models and all other models. Results indicate that the parameters were highly consistent across the population, and even more within each participant (Figure S5, top-left). Next, for each parameter (e.g., one entry in the connectivity matrix), we calculated its intraclass correlation coefficient (ICC), which is the correlation of its value between the two sessions across all participants. ICC characterized the reliability of the individual differences in each parameter.

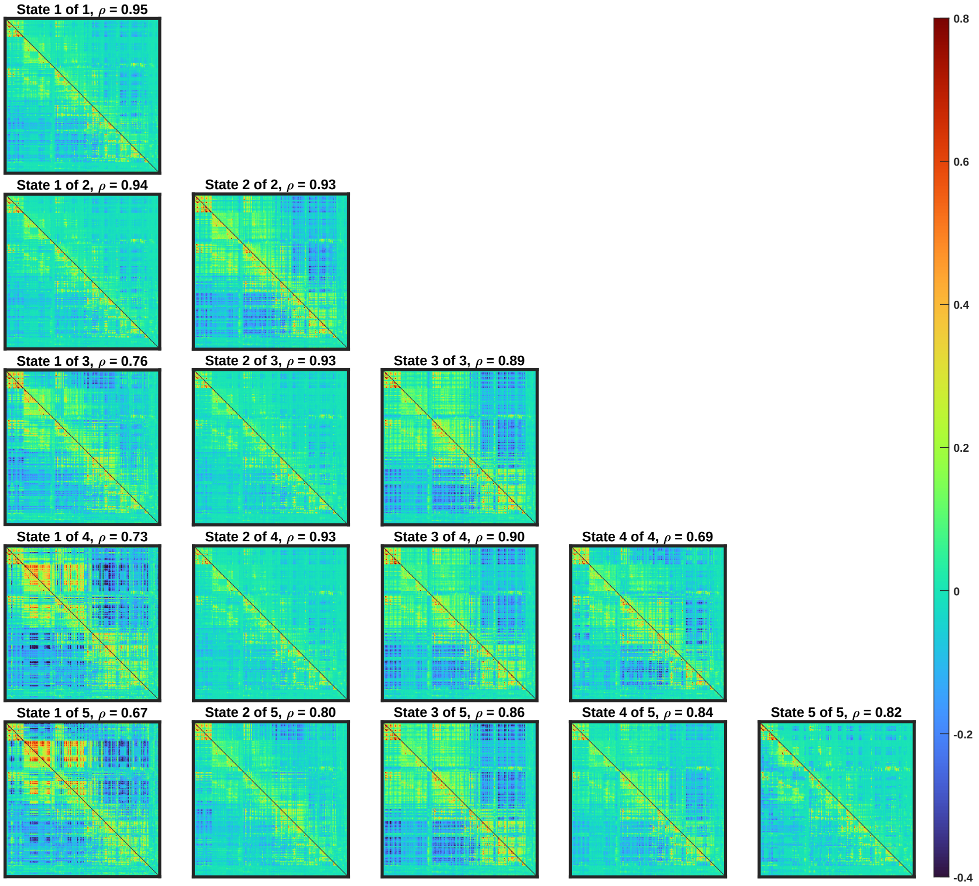

Figure S4: **tvFC clusters from data and MINDy simulation.** Top to bottom rows correspond to solutions with one to five clusters. Each panel shows one cluster centroid (i.e., one tvFC 'state'). The lower triangle is obtained using the deconvolved rfMRI data and upper triangle is obtained from noisy simulations of MINDy models. Values in the panel title indicates correlation between the two.

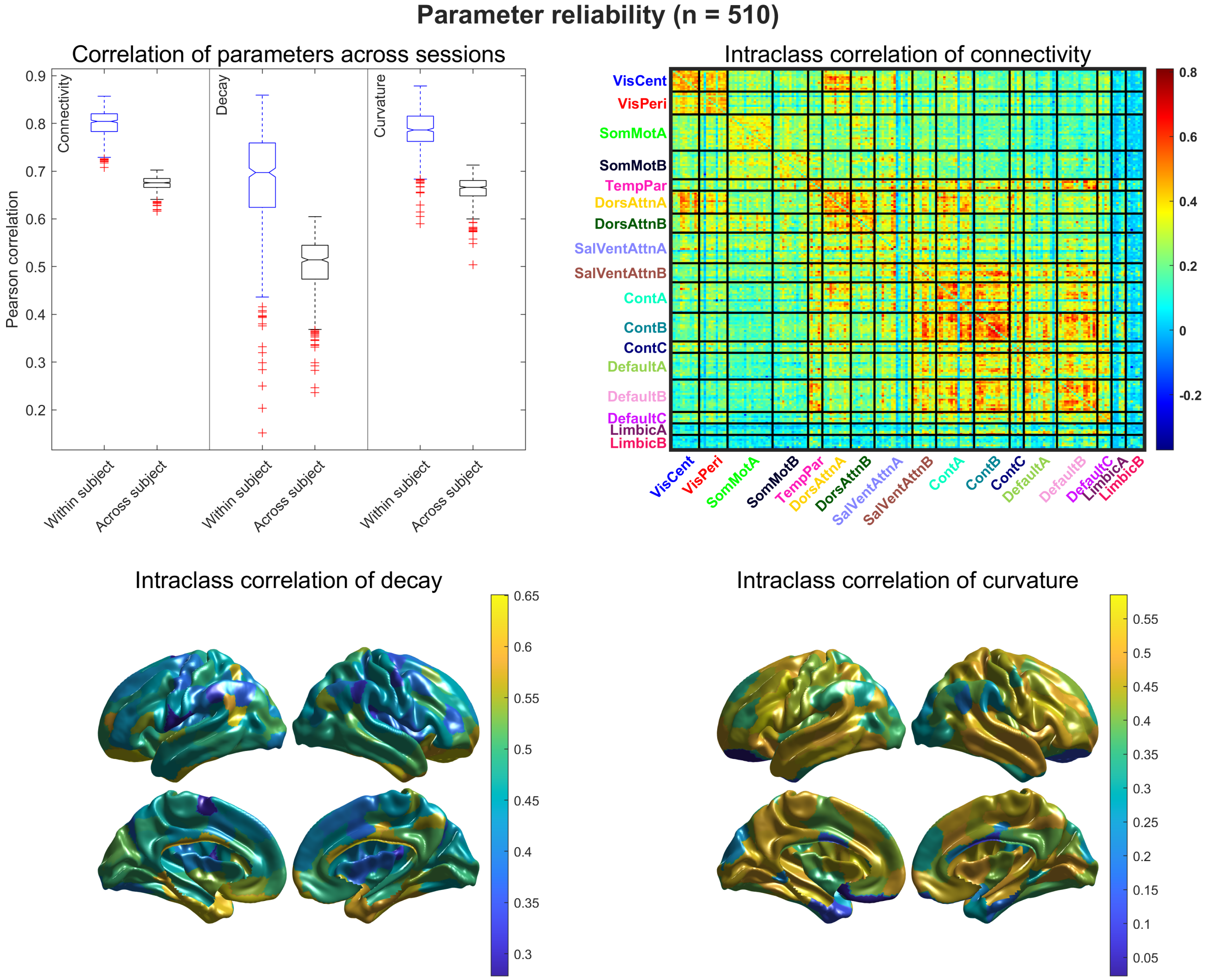

Figure S5: **Obtained parameters were individualized and consistent across the population.** Top-left: Correlation between sets of parameters (connectivity, curvature or decay) from models from different sessions. 'Within subject' is the correlation between the two models from a same participant, while 'across subject' is the mean similarity with all other models. Top-right: intraclass correlation coefficient (ICC) of each connectivity parameter. Text labels indicate the functional networks, separated by the black thick lines. Bottom: ICC for decay and curvature parameters, respectively.

We observed a large set of connectivity parameters with high ICC (Figure S5, top-right; also note that the connectivity is sparse so a lot of entries have low ICC). The ICC for curvature and decay parameters were acceptable, around 0.5 (Figure S5, bottom).

2.2 MINDy parameters encode cognitive differences

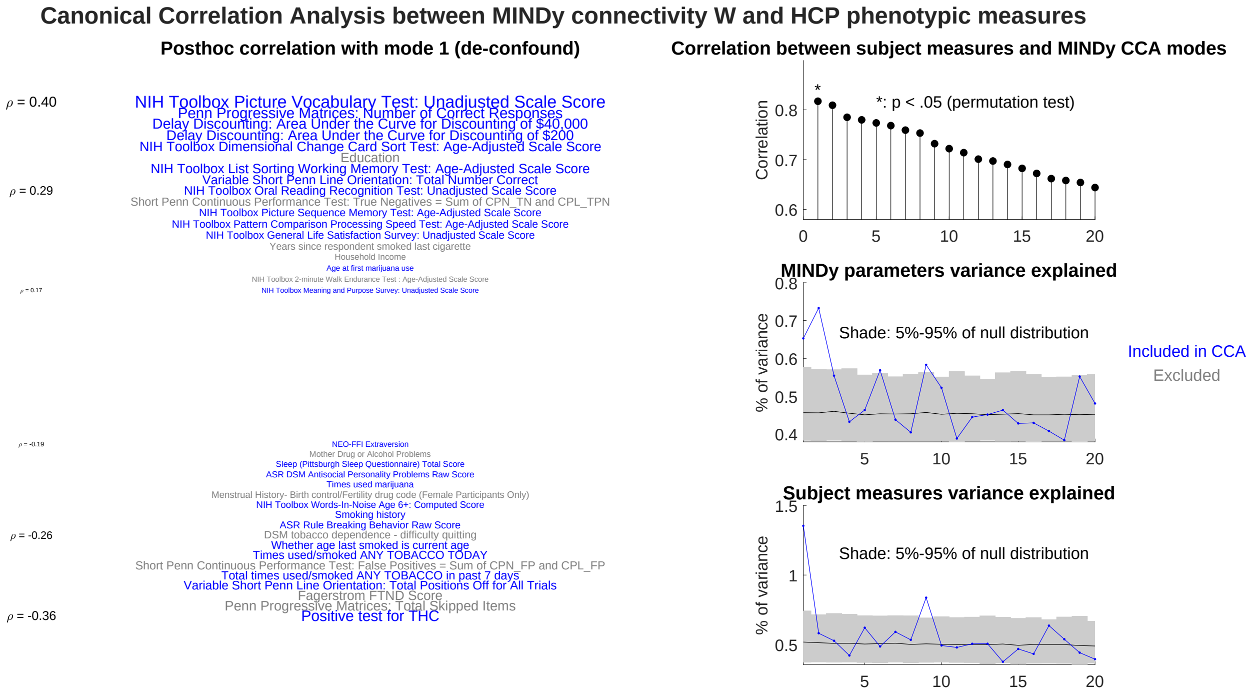

Figure S6: **MINDy parameters encoded reliable individual differences.** Please compare with Figure 1 in (Smith et al., 2015). Left: Post-hoc correlation between the behavioral mode identified by CCA and the phenotypic measures. We listed the most correlated measures with font size scaled by the correlation. Note that the Y axis is ordinal but not scalar. Top-right: correlation between CCA-identified connectivity and behavioral modes. Statistical significance is determined by permutation test with 1000 permutations (same for other panels). Mid-right: Variance of the connectivity explained by the CCA modes. Shaded region indicates the null distribution with 1000 permutations. Lower-right: variance of the behavioral measures explained by the CCA modes.

Here, we conducted a canonical correlation analysis (CCA) between the connectivity matrices of fitted models and the phenotypic measures in the HCP. The connectivity matrices from the two sessions were averaged within each participant before entering the analysis. We used the scripts provided by (Goyal et al., 2020) which extends (Smith et al., 2015) to the whole HCP dataset. The connectivity matrices and subject measures were first projected to their first 100 principal components to reduce dimensionality.

Then, CCA was carried out to identify the directions to which the projection of the connectivity data and phenotypic data are maximally correlated across the population. We identified a unique pair of such directions (modes) with statistically significant correlation (1000 permutations, Figure S6, top right). Further, this connectivity mode and phenotypic mode explained a significant amount of variance in their data respectively (Figure S6, middle and bottom right). Post-hoc correlation between the phenotypic mode and all phenotypic measures revealed that this mode is most related to fluid intelligence and substance use (Figure S6, left). Interestingly, our results are very similar to the original finding in (Smith et al., 2015) even though we are using very different node types (parcels vs. ICA networks) and connectivity measures (effective vs. correlational).

2.3 MINDy connectivity correlates with functional and structural connectivity

Instead of enforcing a set of connection weights based on structural connectivity, MINDy approximates the effective connectivity between brain regions directly from data. Here, we explored whether the MINDy effective connectivity matrix W correlates with structural connectivity SC as well as raw functional connectivity (correlation between deconvolved parcel activities) FC. We averaged the FC matrices (Figure S7, top left) and W matrices (Figure S7, top middle) across all sessions. We obtained structural connectivity matrices for the same brain atlas (but from a different population of healthy young adults) from the MICA-MICs dataset (Royer et al., 2022) and again average across all participants to obtain a population level structural connectivity matrix (Figure S7, top right). The MINDy W matrix is strongly correlated with FC but much sparser, and they are both weakly but significantly correlated with SC (Figure S7, bottom row).

3 Numerical analysis of attractors

3.1 Examples of models that were excluded due to numerical issue

We visually inspected the PCA plots of simulated trajectories and identified 20 (out of 1020) models where numerical identification of attractors seems to have failed. The models from the same participant were also excluded, reducing the number of participants from 510 to 490. Figure S8 showed the simulated trajectories and results of numerical analysis in some of the censored models.

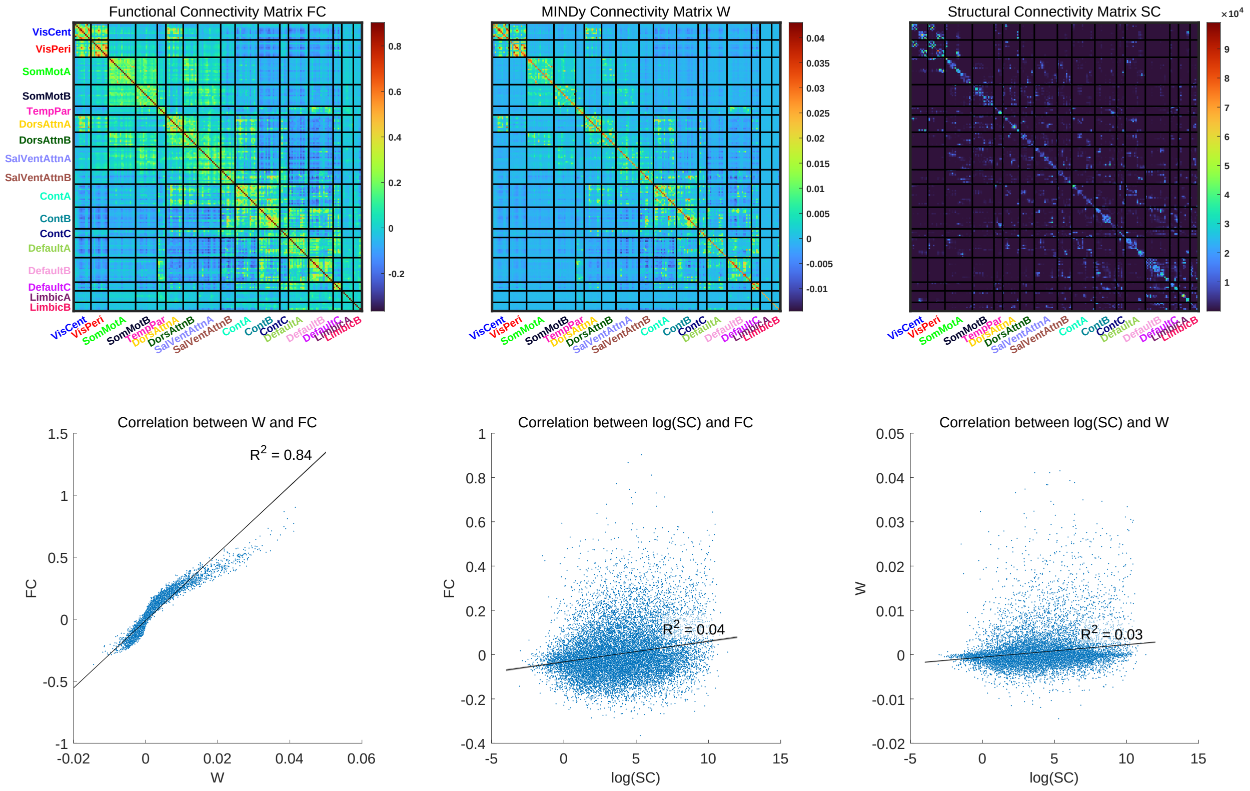

Figure S7: **Comparison between MINDy effective connectivity, functional connectivity, and structural connectivity.** Top row, left to right: Population-averaged functional connectivity matrix FC, MINDy W matrix, and structural connectivity matrix SC. Structural connectivity between two parcels is defined as the total number of DTI streamlines between them. Bottom row, left to right: correlation between off-diagonal entries of the three matrices. Left: correlation between W and FC. Middle: correlation between $\log(SC)$ and FC. Right: correlation between $\log(SC)$ and W.

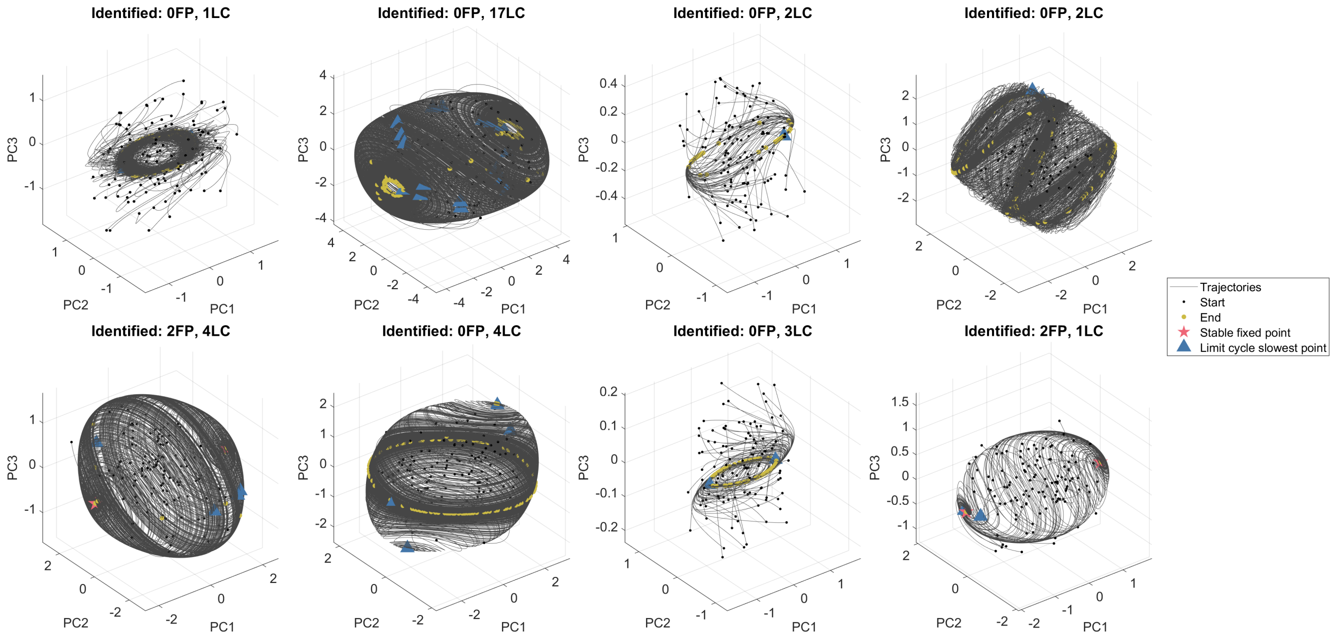

Figure S8: **Example of models excluded from further analysis.** Panel title indicates the number and types of attractors identified by the numerical procedure.

3.2 Influence of different initial conditions

Here we show that the extracted attractors are the same regardless of whether we initialize the simulations using 120 samples from standard normal distribution or 1000 samples from deconvolved rfMRI data. On all 980 fitted models, we compared the number and type of attractors extracted using the two different methods and obtained identical result in more than 99% of models (Figure S9 left). In these models, we paired the extracted equilibria and ghost attractors using Hungarian algorithm with the Euclidean distance as the cost. We normalized the mean distance between paired attractors by the mean Euclidean norm of the attractors. The histogram was plotted in Figure S9 and the distance was extremely small for most models, indicating that the numerical procedure was reliable using only 120 random samples.

4 Taxonomy of model dynamics

4.1 Distribution of the type of dynamics across models

In Figure S10 we show the distribution of the type of dynamics (number and type of attractors) observed in all models without numerical issues (1000 out of 1020).

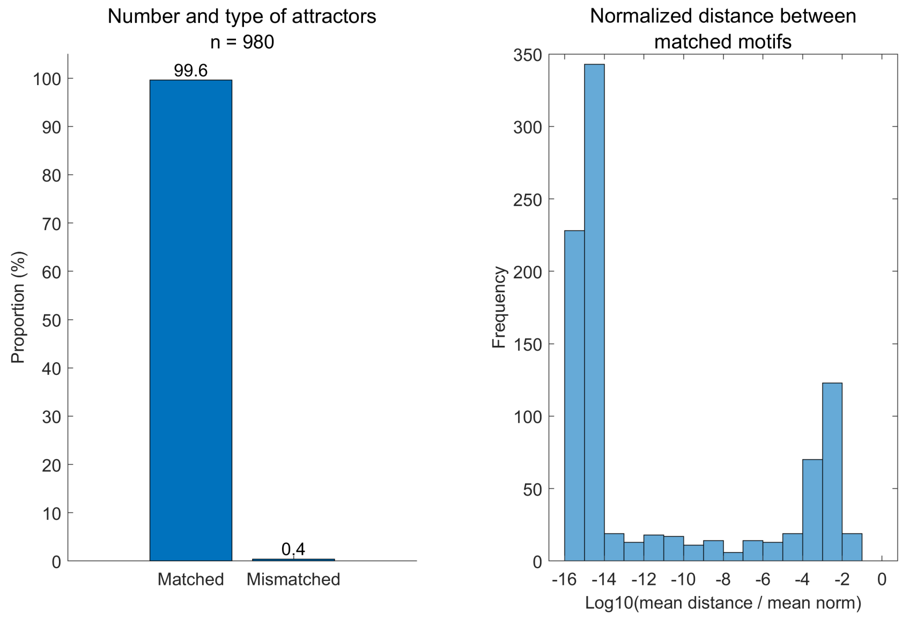

Figure S9: **Comparison between extracted attractors using two different initialization methods.** Left: Proportion of models showing matched or mismatched number and type of attractors using either 1000 random frames from deconvolved data, or 120 random samples from standard normal distribution. Right: Histogram of normalized mean distance between extracted motifs using two different initialization methods.

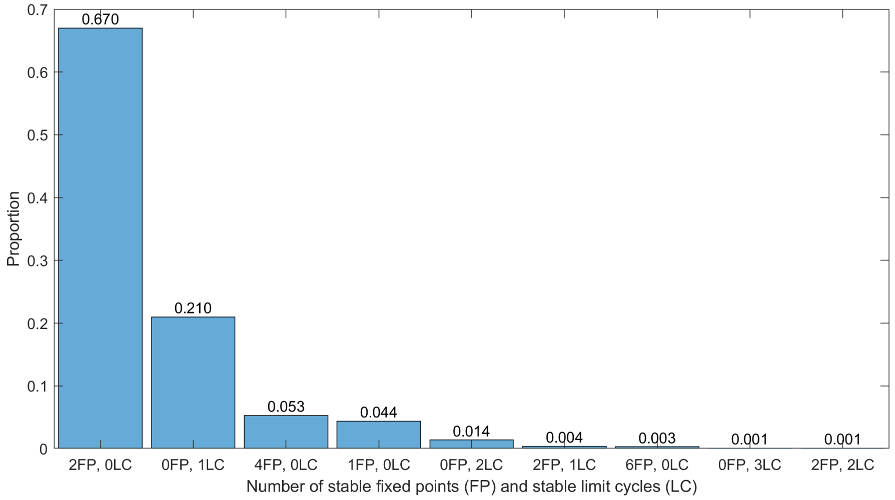

Figure S10: **Taxonomy of dynamics in fitted models.** ‘FP’ indicates stable equilibria and ‘LC’ indicates stable limit cycles.

4.2 Examples of less frequently observed dynamics

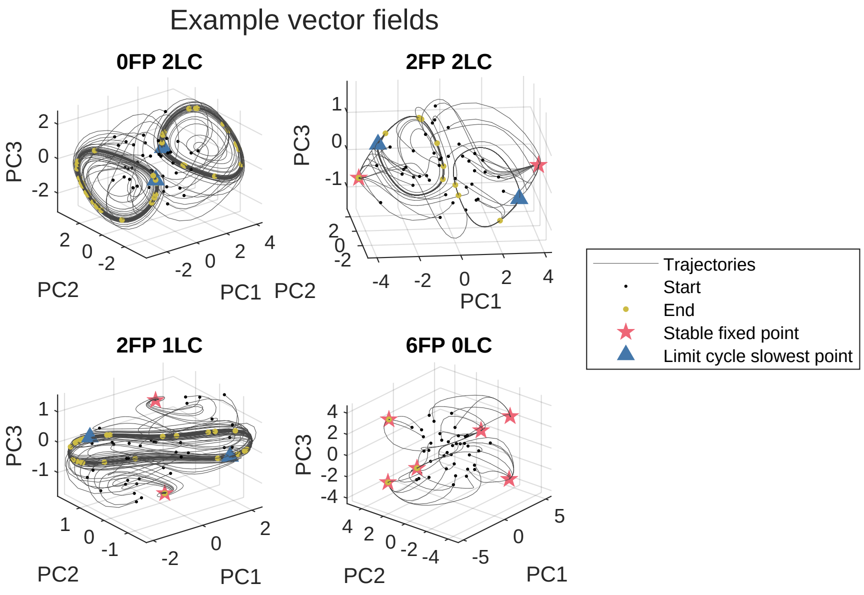

Figure S11: **More examples of dynamical landscapes.** See Figure 1 in main text.

In Figure S11 we show more examples of the dynamics observed in the fitted models. The three except the top-left one were grouped into ‘others’ type in the main text because of their rare occurrence.

5 Toy model for infinite-period bifurcation

Here we show a toy model for infinite period bifurcation mentioned in the main text. The model dynamics are written in polar coordinates (but shown in Cartesian coordinates in Figure S12) as:

$$\begin{cases} \dot{r} = r(1 - r^2) \\ \dot{\theta} = \mu - |\sin\theta| \end{cases} \tag{1}$$

where μ is the bifurcation parameter. The infinite-period bifurcation happens when μ equals one. When $\mu > 1$, the system shows a stable limit cycle and an unstable equilibrium at the origin. As μ approaches 1, the speed distribution on the limit cycle becomes more and more extreme and a ghost attractor emerges at $(0, 1)$ (as well as $(0, -1)$) on the limit cycle. When μ equals 1, the ghost attractor dissolves into a pair of equilibria, one stable and one unstable.

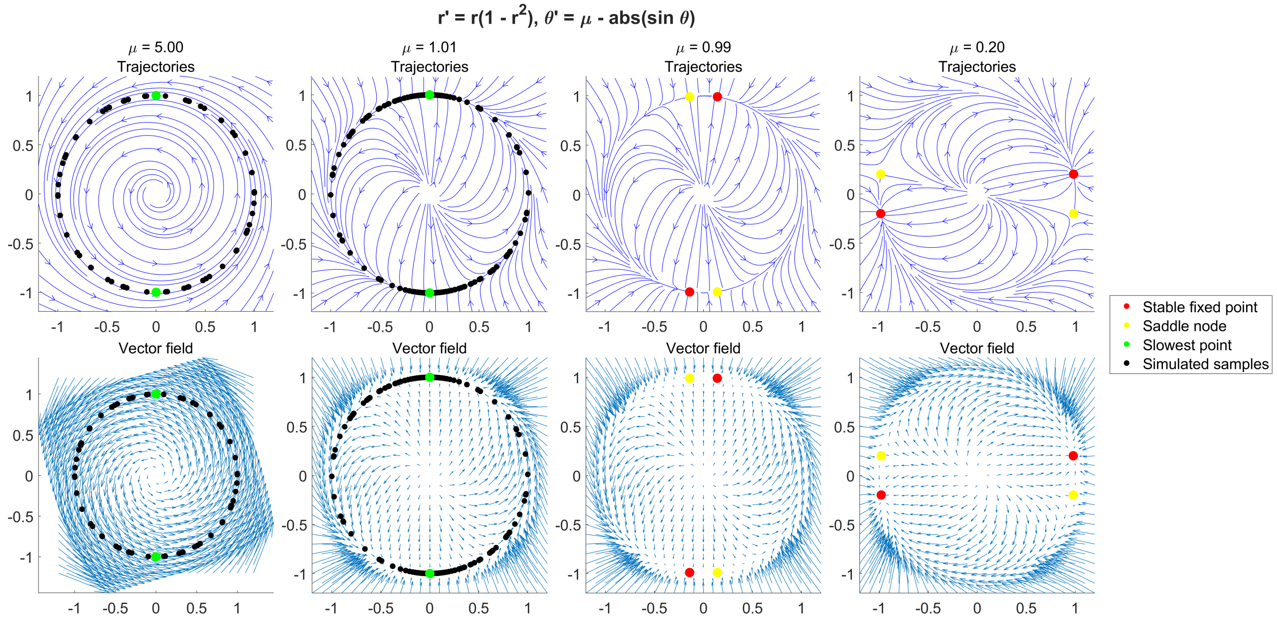

Figure S12: **Toy model for infinite period bifurcation.** Top row: trajectories of the models with different value for the bifurcation parameter μ. Red, yellow and green dots indicate stable equilibria, saddles and the slowest points on limit cycles respectively. Black dots are the simulated samples on the limit cycle. Bottom row: vector fields of the models.

6 Clustering of attractor patterns

6.1 Selection of the number of clusters

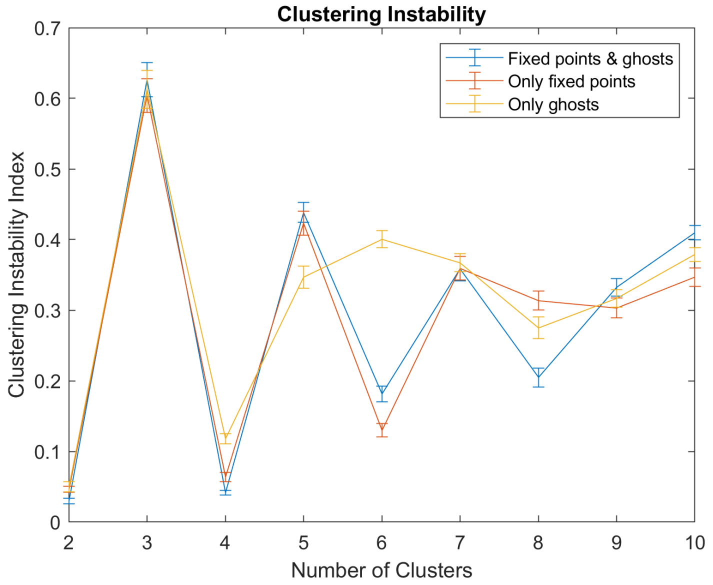

Figure S13: **Stable clustering solution for two and four clusters.**

We used K-means algorithm to cluster the attractors and select the number of clusters K using the cluster instability index (see Methods for more details). We found that regardless of whether we use only the stable equilibria, only the ghost attractors, or both, we got near perfect clustering stability only for K equals to two or four (Figure S13).

6.2 Two cluster solution

We show the two-cluster solution in Figure S14. In this case the two clusters are the reflections of each other. One of them showed strong activation for the DMN and FPN, while the other show strong activation for the visual and the dorsal/ventral attention networks.

6.3 Clustering results with only stable equilibria or only ghosts

In the main text we clustered all stable equilibria and ghost attractors together. Here we show the results using only stable equilibria (Figure S15) and only ghost attractors (Figure S16) respectively.

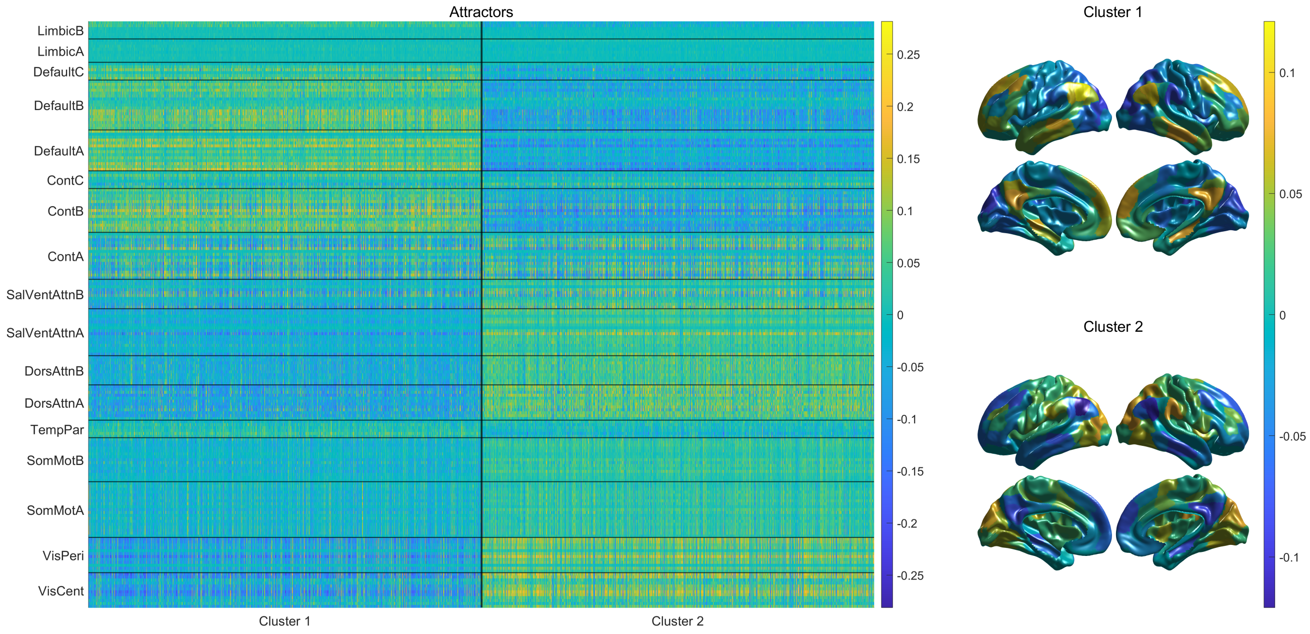

Figure S14: **Two-cluster solution of the attractor clustering.**

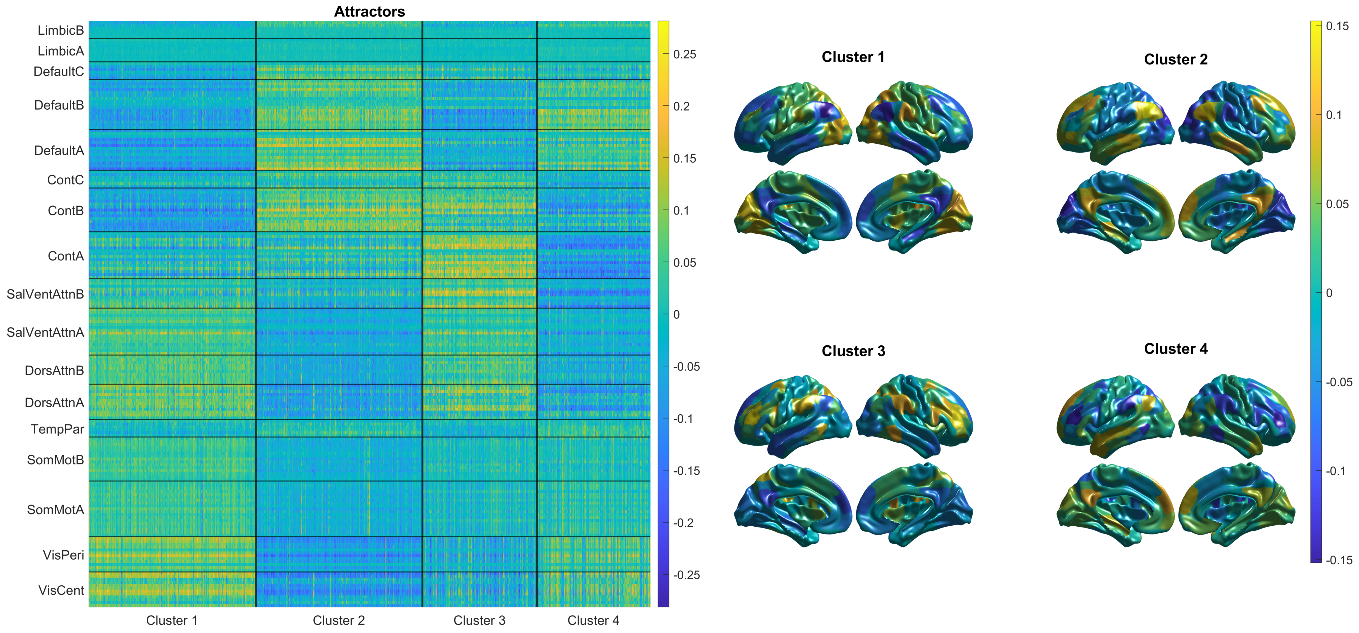

Figure S15: **Attractor clustering with only the stable equilibria.**

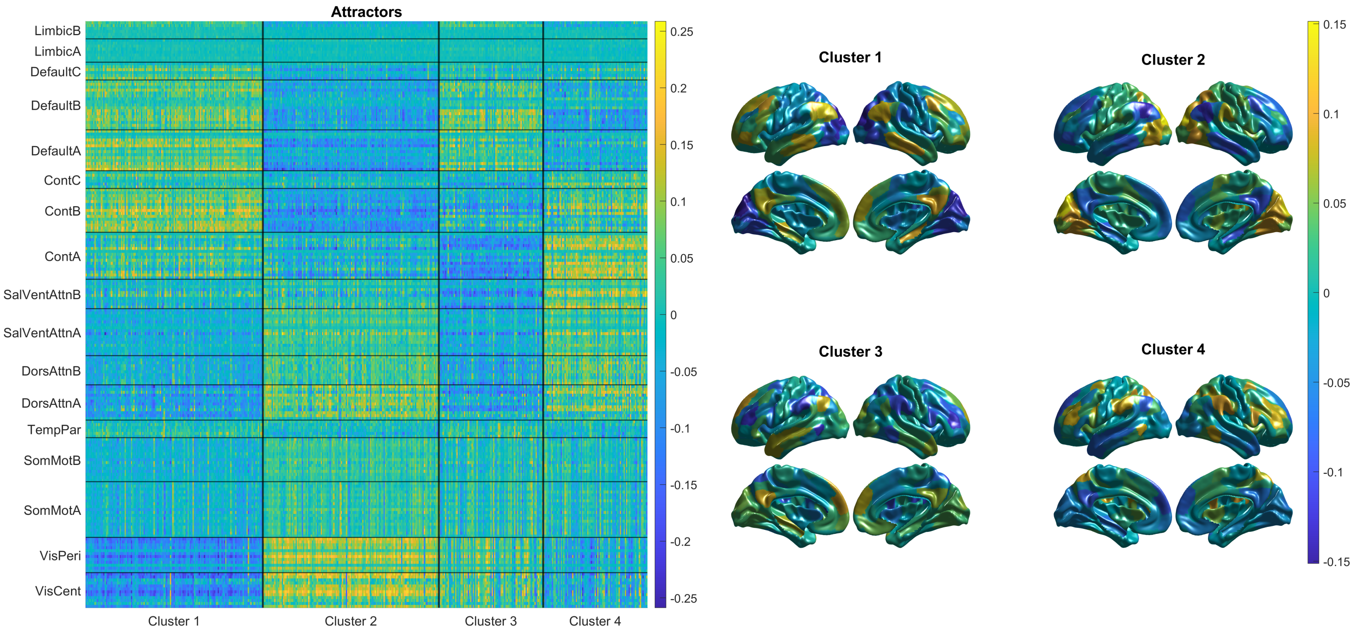

Figure S16: **Attractor clustering with only the limit cycle slowest points.**

6.4 Clustering results with robust sparse K-means algorithm

We also ran the clustering analysis with the robust sparse K-means algorithm which is more robust against outliers (Brodinová et al., 2019). Results are shown in Figure S17. This algorithm contains a hyperparameter for sparsity level s ranging from 1 to $\sqrt{n}$ where n is the dimension of the data. We identified the optimal s using the `wrskGap()` function in the `R` package `wrsk` provided in the paper. Across a wide range of s, the algorithm identified only a small proportion of outliers (with weights smaller than 0.5), and the cluster centroids were nearly identical with the ones obtained through standard K-means (Figure 4).

7 Parcel activation in attractors

7.1 Parcel activation follows network and cluster structure

We analyzed the activation of parcels across all attractors with a mixed-effect model: *activation* $\sim$ *1* $+$ *cluster* $+$ *network* $+$ *cluster:network* $+$ *(cluster*$|$*network:parcel)*. We computed the hierarchical (type I) sum of squares explained by each term (Figure S18). The main effects of cluster and network were small while their interactions explained over 40% of total variation, indicating that (1) the same brain network

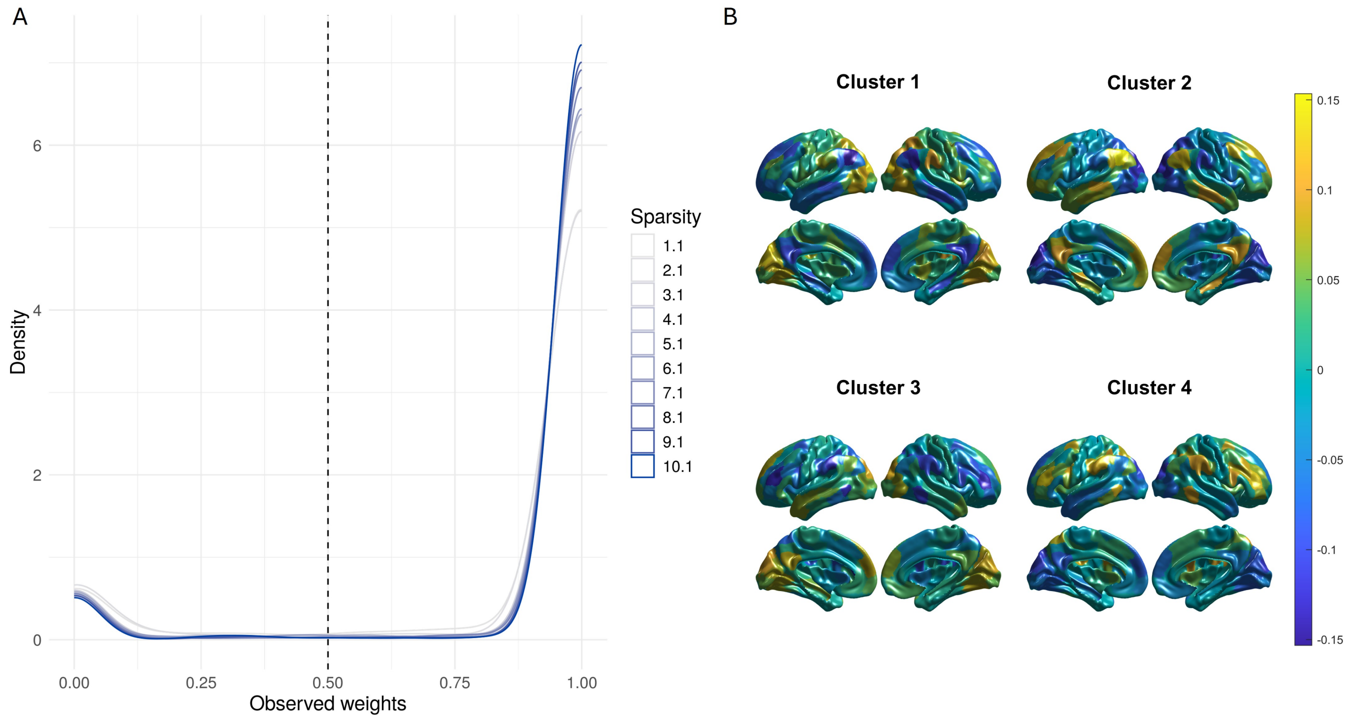

Figure S17: **Clustering results with robust sparse K-means algorithm.** Left: distribution of computed weights for each observation (attractor). Each line represents the results using one sparsity parameter as indicated by the legend. Dash line indicates the threshold (0.5) for outliers detection. Observations with weights smaller than this threshold were considered outliers. Right: cluster centroids identified.

showed very different activation in different clusters (or equivalently, that each cluster is associated with the strong activation of different sets of networks); and (2) parcel activation was mostly determined by the combination of attractor clusters and functional brain network structure. The random effect of parcels explained about 15% of variation, indicating that heterogeneity still exist across the parcels within each network. The error sum of squares was smaller than the total variation explained by the modeling, suggesting high consistency across participants and sessions.

7.2 Functional rather than spatial organization explains data better

To show that the activation was driven by functional network segmentation rather than the spatial proximity between parcels, we calculated the Pearson correlation between the activation patterns of each pair of parcels across all attractors. The correlation coefficient was Fisher-transformed into a Zr statistic (Figure S19, top-left). We modeled this similarity matrix by the combination of the spatial proximity (negative cortical distance) between parcels and their functional network assignment. The distance between all cortical vertices along the surface were calculated using the surface geometry file from HCP and MATLAB's graph distance function, and then averaged within the two parcels under consideration. All inter-hemisphere entries were excluded since the distances were undefined. The functional network assignment similarity was set to one if two parcels belong to the same network as defined by the 17-network atlas in (Schaefer et al., 2018), and zero otherwise. We then predicted the activation similarity using negative cortical distance, functional network assignment and their interactions. A hierarchical sum of squares analysis showed that network organization explained 15% of total variation even after excluding the effect of spatial proximity (which explained must less variance, Figure S19, bottom-right, first column). Therefore, the attractors indeed reflected the organization of functional brain networks over and above the spatial configuration of cortical regions.

8 Results without two-point derivative smoothing

In the main text we estimated the derivative of the dynamics using two-point forward differences $\hat{\Delta}\mathbf{x}_n = (\mathbf{x}_{n+2} - \mathbf{x}_n)/2$. This effectively doubled the sampling interval to 1.44 seconds, which is closer to the TR used in most non-HCP studies. Here we repeated the analysis on a subset of 100 participants without this two-point smoothing step, i.e., $\hat{\Delta}\mathbf{x}_n = \mathbf{x}_{n+1} - \mathbf{x}_n$. The taxonomy of observed dynamics was shown in S20. The most popular types of dynamics were still two equilibria or one limit cycle. The clustering instability index for clustering (ghost and point) attractors were shown in Figure S21 and suggested

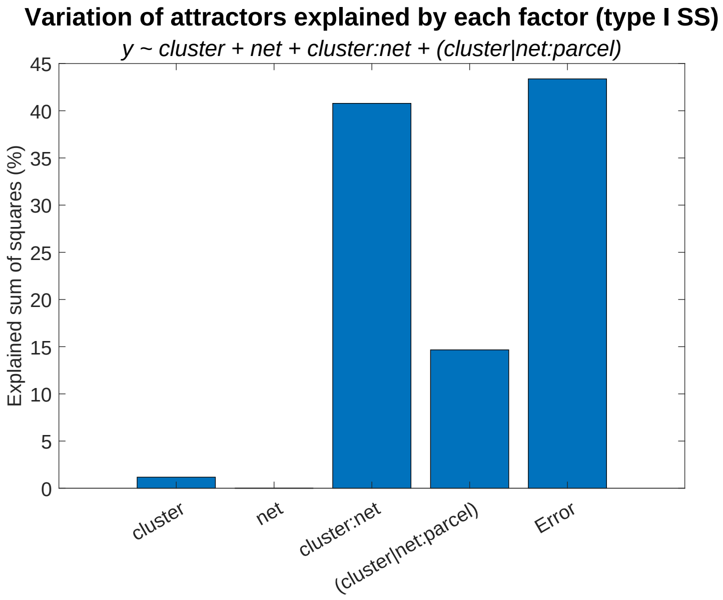

Figure S18: **Variance of parcel activation explained by attractor clusters and functional networks.**

similar preferences for two-cluster and four-cluster solutions. The four-cluster solution was shown in Figure S22 and the emerged cluster centroids were also very similar to the results in the main text.

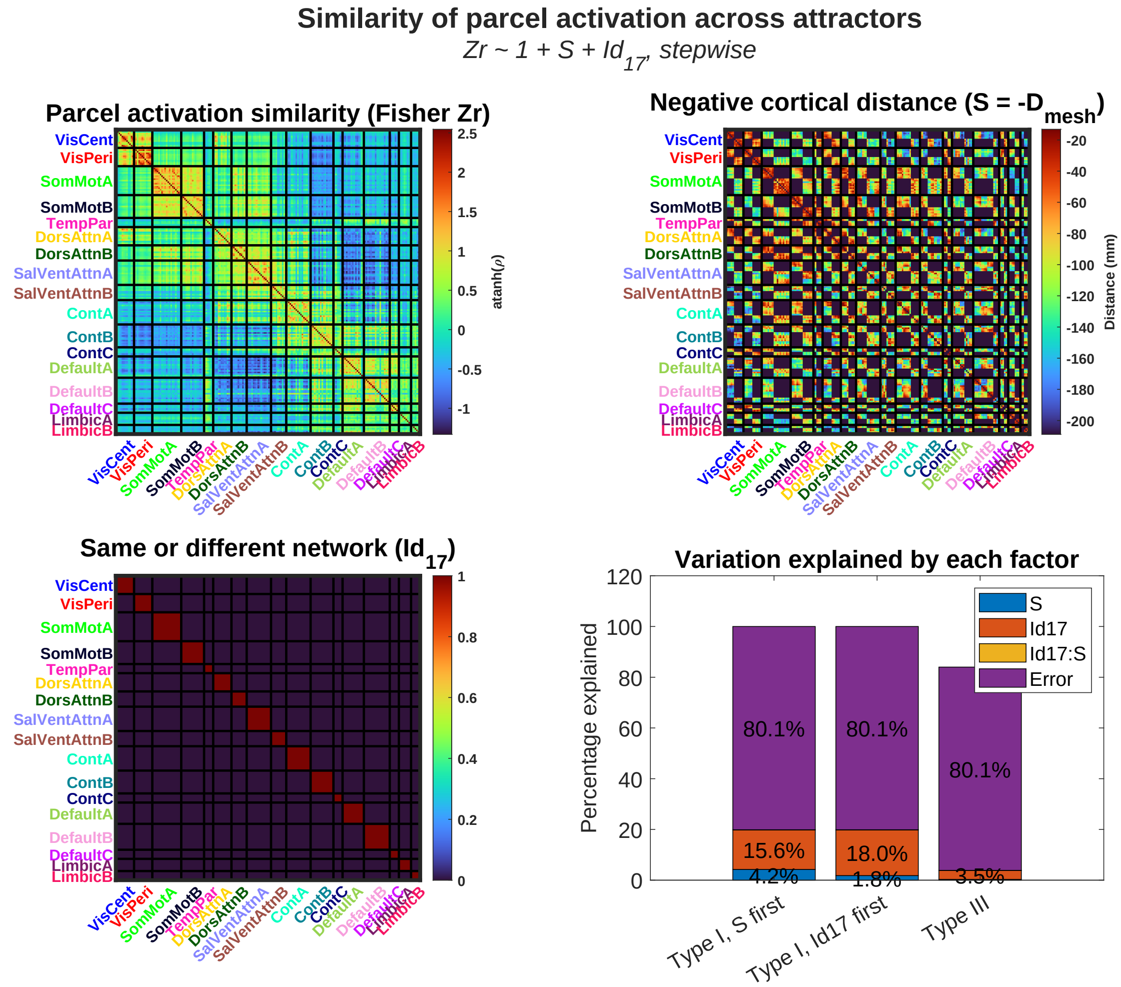

Figure S19: **Parcel activation similarity across all attractors.** Top-left: similarity between all parcels' activation across all attractors. Similarity is quantified by Pearson correlation followed by Fisher's transformation. Top-right: the negative of the distances between parcel centroids along the cortical surface. Inter-hemisphere entries were omitted. Bottom-left: similarity between parcels based on (Schaefer et al., 2018) functional network segmentation. Similarity is one if the two parcels belong to the same network and zero otherwise. Bottom-right: variance of parcel activation similarity explained by distance or network structure. Column one: type I (hierarchical) sum of squares (SS) where cortical distance precedes network structure (see the text). Column two: type I SS where network structure precedes cortical distance. Column three: type III SS. The SS for the interaction between distance and network structure is less than 1%.

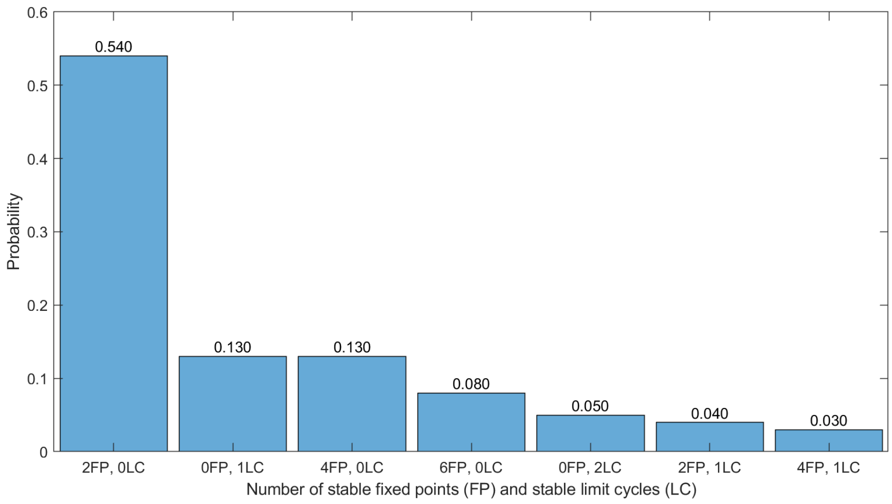

Figure S20: **Taxonomy of dynamics in models fitted with one-step instead of two-step difference.** 'FP' indicates stable equilibria and 'LC' indicates stable limit cycles.

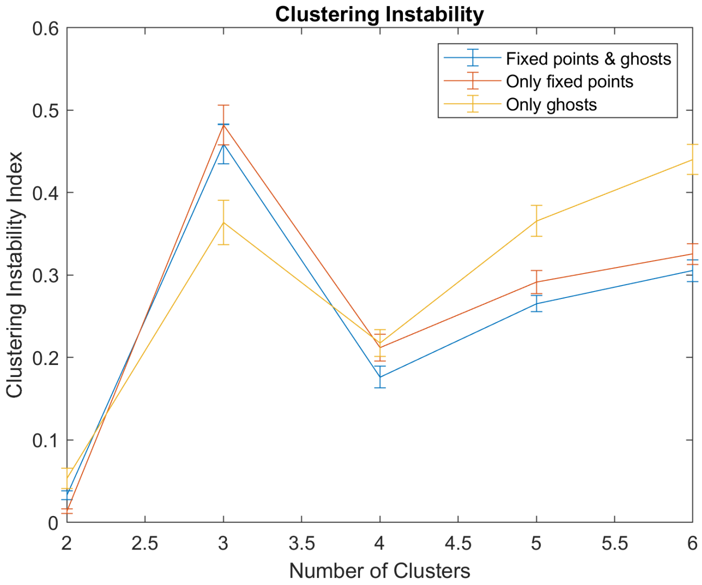

Figure S21: **Stable clustering solution for two and four clusters in one-step difference models.**

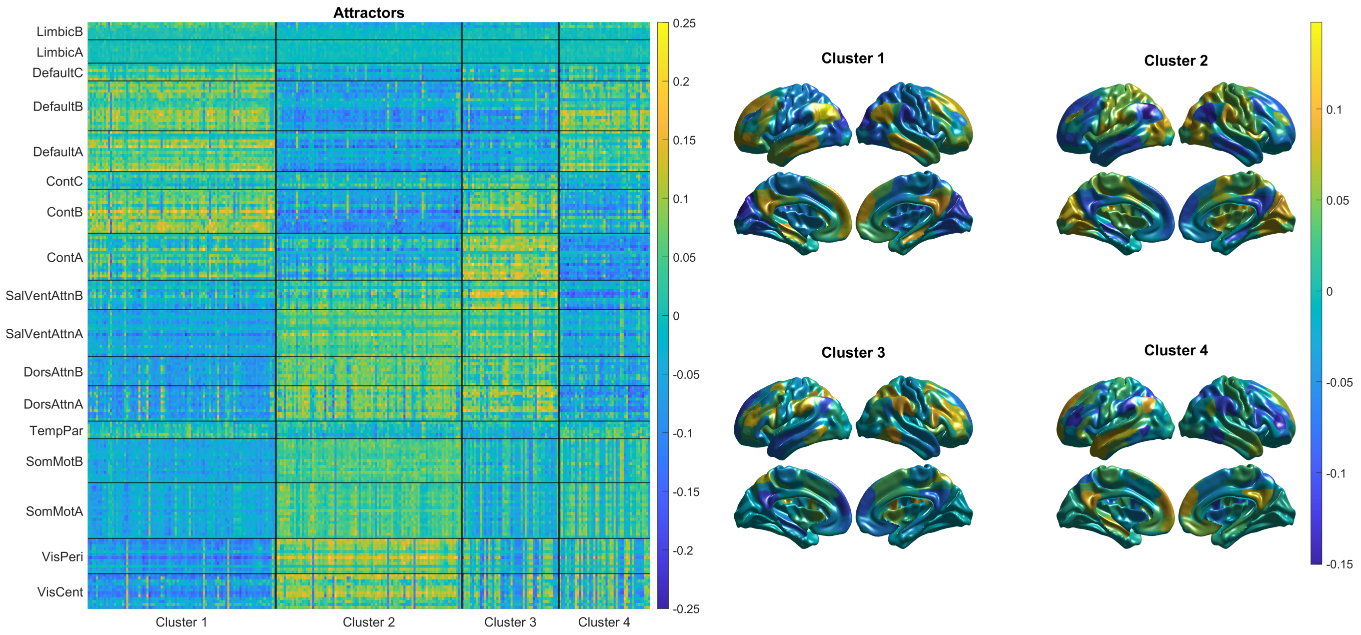

Figure S22: **Clustering (ghost and point) attractors from one-step difference models.**

9 Supplementary videos

We simulated four obtained models with different amount of additive Gaussian white noise, and visualized the trajectories in the Supplementary videos. For each model, we first obtained the attractors using 120 noise-free simulations, and calculated the first two principal components (PCs) of the noise-free simulations. The attractors were projected to the two PCs and shown in the left panel of the video. We then simulated 30 trajectories of the models under certain amount of noise. The states from all 30 noisy simulations were visualized as a gray scatter plot on the left panel. We selected one of the trajectories and showed the evolution of the state along this trajectory frame-by-frame, denoting the current state as a green hexagram projected onto the PCs. Meanwhile, we also visualized the state as a 200-dimensional parcel activation pattern (as in Figure 4) over the cortex on the right panel. The title indicates current simulation time steps (TRs).

1. `PCSurf_dW_00_0FP_1LC.mp4`: a model with one stable limit cycle, simulated without noise. Note the state changed rapidly when traversing the bottom and top of the limit cycle, but it changed very slowly around the left and right extremes.

2. `PCSurf_dW_00_2FP_0LC.mp4`: a model with two stable equilibria, simulated without noise.

3. `PCSurf_dW_10_2FP_1LC.mp4`: a model with two stable equilibria and one stable limit cycle,

simulated with Gaussian white noise with standard deviation 0.1 (i.e., one-tenth of the scale of the training data). Note that depending on which region in the state space the state was traversing, the parcel activation pattern sometimes showed synchronized oscillation (near the limit cycle) and sometimes desynchronized fluctuations (near the equilibria).

4. `PCSurf_dW_20_4FP_0LC.mp4`: a model with four stable equilibria, simulated with noise with standard deviation 0.2. Note how the state lingered around one stable pattern for a while before switching to other patterns under the influence of noise.
